# Regional diffusion imaging measures to disentangle SVD-related hypertensive arteriopathy versus cerebral amyloid angiopathy

**DOI:** 10.1186/s13024-026-00942-4

**Published:** 2026-04-13

**Authors:** Sheelakumari Raghavan, Scott A. Przybelski, Robel K. Gebre, Audrey Low, Mingzhao Hu, Robert. I. Reid, B. Gwen Windham, Heather J. Wiste, Angela J. Fought, Michael G. Kamykowski, Aivi T. Nguyen, Melissa E. Murray, Val J. Lowe, Clifford R. Jr Jack, Ronald C. Petersen, Jonathan Graff-Radford, Prashanthi Vemuri

**Affiliations:** 1 Department of Radiology, Mayo Clinic, 200 First Street SW, Rochester, MN 55905 USA; 2 Department of Quantitative Health Sciences, Mayo Clinic, Rochester, MN 55905 USA; 3 Department of Information Technology, Mayo Clinic, Rochester, MN 55905 USA; 4 Department of Medicine, The MIND Center, University of Mississippi Medical Center, Jackson, FL 39216 USA; 5 Department of Laboratory Medicine and Pathology, Mayo Clinic, Rochester, MN 55905 USA; 6 Department of Laboratory Medicine and Pathology, Mayo Clinic, Jacksonville, FL 32224 USA; 7 Department of Neurology, Mayo Clinic, Rochester, MN 55905 USA

**Keywords:** Diffusion MRI, Small vessel disease, Hypertensive arteriopathy, Cerebral amyloid angiopathy, Pathology, Cognition

## Abstract

**Background:**

Detecting and distinguishing early changes due to the two key subtypes of cerebral small vessel disease (SVD), hypertensive arteriopathy (HA) and cerebral amyloid angiopathy (CAA), has significant clinical implications. Our goal was to develop and validate dMRI signatures associated with HA-SVD and CAA-SVD proxies and assess their clinical utility using Alzheimer’s disease and SVD biomarkers, pathology, and cognition.

**Methods:**

Two independent cohorts with baseline dMRI scans, T2* gradient-echo MRI, and vascular risk measures were analyzed: Mayo Clinic Study of Aging (MCSA, *N* = 1080) and Alzheimer’s Disease Neuroimaging Initiative (ADNI, *N* = 549). In MCSA, regional dMRI measures associated with proxies of HA-SVD (hypertension) and CAA-SVD (lobar cerebral microbleeds) were identified using logistic regression models. The top regional features were then used to compute composite dMRI indices for HA-SVD and CAA-SVD. These dMRI indices were validated in ADNI and in an independent pathology sample of MCSA (*N* = 147). In MCSA, we also computed standard global SVD indices from diffusion and FLAIR MRI and compared them with dMRI indices to reflect SVD subtypes. Next, we evaluated the association of these indices with cognitive performance (global, attention, and memory) using regression models, after adjusting demographics, white matter hyperintensities (WMH) and amyloid.

**Results:**

Hypertension was associated with reduced microstructural integrity predominantly in fronto-parieto-projection pathways, whereas lobar microbleeds were associated with occipito-parietal damage. These differential tract association patterns with HA-SVD and CAA-SVD proxies were less pronounced in ADNI. In the community-dwelling MCSA cohort with higher prevalence of vascular disease, dMRI indices provided more differentiated associations with both proxies of SVD than global SVD indices, underscoring added value for etiology-specific identification. As expected, CAA-SVD indices were more strongly associated with occipital WMH and amyloid burden and were linked to CAA pathology scores. CAA-SVD indices also had greater association with memory performance, independent of amyloid and WMH. Conversely, HA-SVD indices were robustly associated with post-mortem Kalaria scales and attention scores.

**Conclusions:**

Using three datasets, (population-based sample, independent cohort, pathology sample), we found that regional dMRI signatures can capture distinct SVD processes of HA and CAA. These dMRI signatures offer potential for early differential identification of SVD subtypes and can aid in guiding clinical decision making and prevention.

**Supplementary Information:**

The online version contains supplementary material available at 10.1186/s13024-026-00942-4.

## Background

Cerebral small vessel disease (SVD) is a major contributor to vascular cognitive impairment and dementia (VCID) in aging populations [1], causing approximately 25% of ischemic stroke and occurring in about 50–60% of people aged ≥ 65 years. The two most common causes of SVD are arteriolosclerosis (thickening of vessel wall and stenosis of arterioles), primarily contributed by hypertensive arteriopathy (HA), and cerebral amyloid angiopathy (CAA), characterized by amyloid-β deposition in the cortical and leptomeningeal vessels [2]. Both etiologies HA-SVD and CAA-SVD impact small vessel compliance. While other vascular risk factors (VRFs) including diabetes, hyperlipidemia, and smoking may have associations with SVD, the impact of hypertension alone is greatest in randomized controlled trials targeting risk stratification and screening of SVD [3].

MRI markers commonly used to assess SVD burden include white matter hyperintensities (WMH), lacunes, cerebral microbleeds (CMBs), perivascular spaces (PVS) and microinfarcts [4, 5]. CMBs are small hemosiderin deposits detected on T2*-weighted gradient recalled echo sequences or susceptibility weighted MRI that reflect prior microhemorrhage from damaged small vessels [6]. However, the location and distribution of CMBs differ by SVD subtype: deep, infratentorial or basal ganglia lesions are indicative of HA [7], while lobar or centrum semiovale lesions suggest CAA [8]. Composite scores of both HA-SVD and CAA-SVD processes have been developed [9, 10] and modified previously [11] to distinguish between two SVD subtypes. These measures correlate differentially with risk factors [12], inflammation [13], and clinical outcomes, including intracerebral hemorrhage (ICH) [9, 10] and cognitive domains.

While quantification of these etiologies is important for treatment of both SVD and non-SVD etiologies, the visible lesions considered for computing the scores are seen later in the disease process. Identification of early SVD-related changes is an open and critical question to address. This will not only be useful for prevention but also for effectively controlling and mitigating adverse events associated with anti-amyloid therapies, known as amyloid-related imaging abnormalities (ARIA), which occur more frequently in individuals with high SVD-CAA [14]. Additionally, clinical trials that focus on early intervention need biomarkers capable of detecting pathological changes before visible lesions develop.

Given the sensitivity of diffusion MRI (dMRI) measures for capturing microstructural and network disruption due to diffuse SVD well before the appearance of WMH [15, 16], dMRI derived signatures can aid in disentangle the complex profiles of microangiopathies associated with SVD. Although diffusion tensor imaging (DTI) derived metrics such as fractional anisotropy (FA), mean diffusivity (MD), peak width of skeletonized mean diffusivity (PSMD), and free water (FW) have shown promise to independently capture HA and CAA-related changes in antemortem [17–19] and histopathological studies [20, 21], these markers are utilized in the field currently as global measures and therefore non-specific to SVD etiologies. Therefore, the role of regional dMRI signatures to disentangle these etiological subtypes have significant potential. Furthermore, postmortem neuropathological scales such as the Strozyk scale [22], the Kalaria scale [23, 24], and CAA pathology measures offers a validation framework for confirming imaging-pathology correlation [20]. 

In this study, we hypothesized that regional dMRI measures that are sensitive to proxies of HA and CAA will be associated with subtype-specific neuropathology SVD scales and map to etiology specific cognitive domains. To test our hypothesis, we: (i) examined the association between dMRI measures and proxies of HA (hypertension) and CAA (lobar CMBs) in two independent datasets; (ii) derived dMRI signatures or indices from the top regional dMRI features associated with hypertension (HTN) and lobar CMBs, and evaluated their associations with aging and dementia biomarkers; (iii) validated these composite signatures in a non-overlapping well-characterized pathology dataset; and (iv) assessed the usefulness of the composite dMRI signatures in predicting cognitive performance, accounting for imaging biomarkers of Alzheimer’s Disease (AD) and SVD.

## Materials and methods

### Selection of participants

We included individuals, who were at least 50 years of age with baseline dMRI measurements, a baseline T2*-gradient recalled echo imaging, and vascular risk factors (VRFs). Participants included in the study were enrolled in the Mayo Clinic Study of Aging (MCSA; *n* = 1080), an ongoing population-based study of the residents of Olmsted County, MN and the Alzheimer’s Disease Neuroimaging Initiative phase 3 (ADNI; *n* = 549). The Rochester Epidemiology Project (REP) medical records-linkage system was used to enumerate the MCSA sample population [25, 26]. The study design and diagnostic criteria for the MCSA population have been described previously [27]. ADNI is an ongoing neuroimaging research initiative designed to determine whether serial MRI and PET imaging, biological markers, and clinical and neuropsychological evaluations can be integrated to effectively monitor the progression of mild cognitive impairment (MCI) and AD (www.adni-info.org). The MCSA cohort included 996 cognitively unimpaired (CU), 78 participants with MCI, and 4 with AD, and two with missing clinical dementia rating scale information for diagnosis. The ADNI cohort comprised of 307 CU, 183 MCI, and 59 AD participants.

We also identified 147 participants in MCSA with antemortem dMRI scans and two available pathological scales such as CAA and Kalaria scores that can measure extent of SVD damage.

### Standard protocol approvals, registrations, and patient consents

All study procedures were approved by the Mayo Clinic and Olmsted Medical Center institutional review boards and were performed in accordance with the ethical standards of the Declaration of Helsinki and its later amendments. Written informed consent was obtained from all participants/surrogates.

### Demographic and vascular risk profiles

Age and sex were self-reported at the time of the baseline MRI visit and were used as covariates. In MCSA, a composite measure of cardiovascular and metabolic conditions (CMC) was derived based on the number of VRFs (hypertension, hyperlipidemia, cardiac arrhythmias, coronary artery disease, congestive heart failure, diabetes mellitus, and stroke), as previously described [28], and these conditions were abstracted from the healthcare records.

### MR Image acquisition and analyses

All MCSA MR images were acquired from 3T scanners (GE Healthcare, Chicago, IL) using standardized protocols and further analyzed using the inhouse pipelines, as published previously [28–30]. ADNI MR images were also acquired from 3T scanners (GE Healthcare; Siemens; Philips) using previously published harmonized protocols and were analyzed using the same in-house pipelines as the MCSA images [31]. 

### dMRI markers

**Fig. 1:**
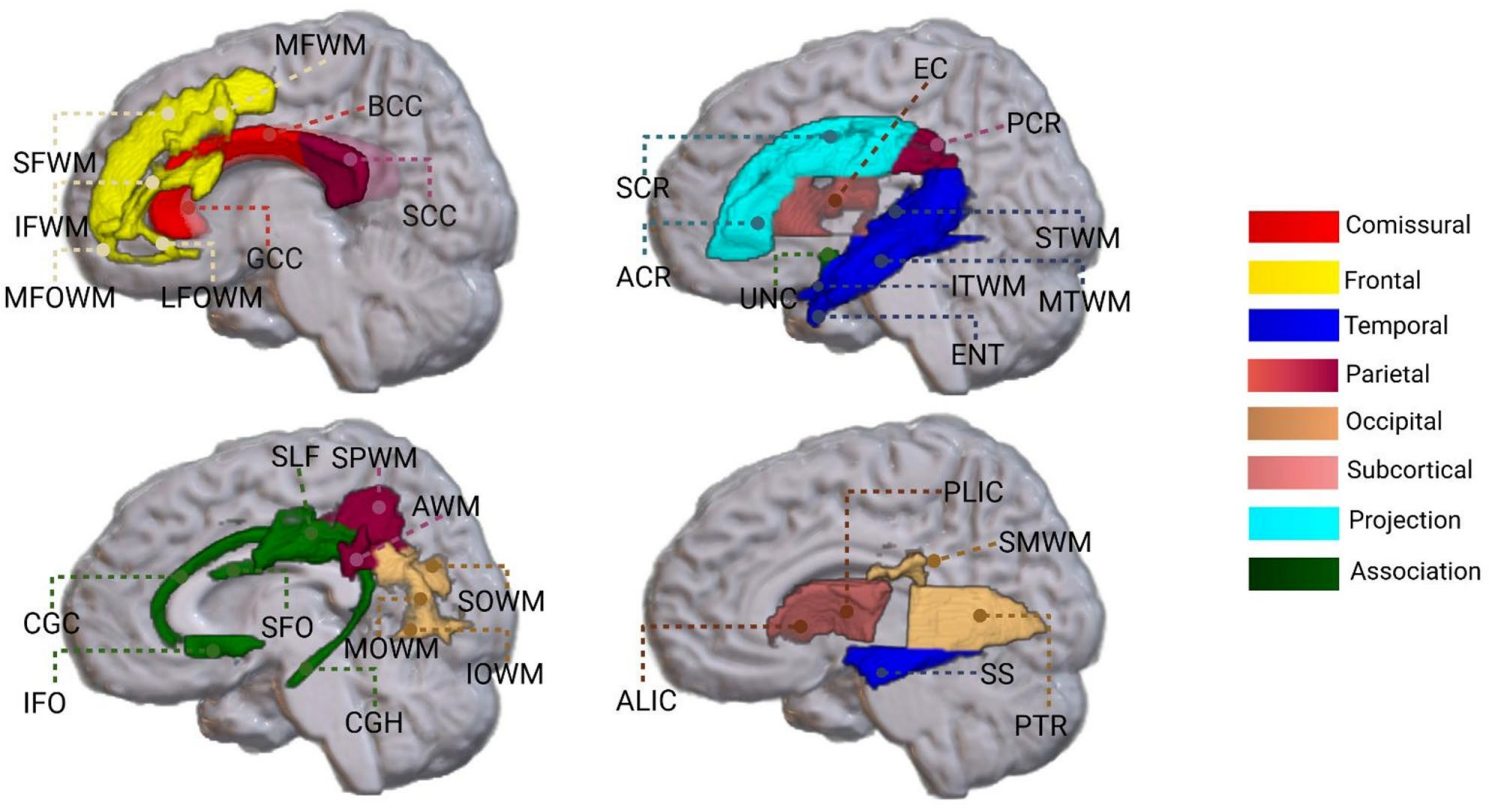
Illustration of the white matter tracts of interest used in the study. Genu, body, and splenium of corpus callosum (GCC, BCC, SCC); inferior, middle and superior frontal white matter (IFWM, MFWM, SFWM); lateral and middle fronto-orbital WM (LFOWM and MFOWM); uncinate fasciculus (UNC); superior fronto-occipital fasciculus (SFO); inferior fronto-occipital fasciculus (IFO); superior longitudinal fasciculus(SLF); cingulum (CGC); parahippocampal cingulum (CGH); anterior, superior, and posterior corona radiata (ACR, SCR, PCR); inferior, middle, and superior temporal WM (ITWM, MTWM, STWM); entorhinal WM (ENT); sagittal stratum (SS); external capsule (EC); anterior and posterior limb of internal capsule(ALIC and PLIC); superior parietal WM (SPWM); angular WM (AWM); supramarginal WM (SMWM); posterior thalamic radiation (PTR); inferior, middle and superior occipital WM (IOWM, MOWM, SOWM)

MCSA dMRI scans were acquired using a spin-echo echo planar imaging sequence with 2.7 mm isotropic resolution. The protocol consisted of five non-weighted images (b = 0 s/mm^2^) and 41 diffusion weighted images (b = 1000 s/mm^2^), with echo time (TE) = 68 ms, and repetition time (TR) = 9951 ms [32]. ADNI 3 protocols have been previously described [33]. Preprocessing steps included denoising [34], correction for eddy current distortion [35], Gibbs ringing [36], skull stripping, and Rician noise bias correction [37]. Diffusion tensors were then fitted to obtain fractional anisotropy (FA) and mean diffusivity (MD) maps using dipy [8]. In order to obtain regional measures, each participant’s FA and MD images were nonlinearly registered to an in-house modified version of Johns Hopkins University (JHU) “Eve” WM atlas using Advanced Normalization Tools software (ANTs). This in-house version was slightly modified by fusing left and right portions of structures spanning the left-right midplane, particularly in the genu and pons. The current study used FA and MD measures from 32 major WM tracts **(**Fig. 1**)**, selected based on prior literature [19, 38]. For MCSA, we also computed the global measures including mean FW fraction and arteriolosclerosis (ARTS) score using MarkVCID scripts [39, 40], while PSMD was calculated using a freely available fully automated script (v 1.8.3 http://www.psmd-marker.com/), as previously published [17]. 

### SVD imaging markers

WMH were segmented on two-dimensional T2 FLAIR using a fully automated algorithm with a custom-made WM mask derived from 3D MPRAGE segmentation [41]. Total intracranial volume (TIV) was calculated using SPM segmentation, and WMH volume was expressed as percentage of TIV. CMBs were identified on T2* GRE scans in both cohorts and subsequently confirmed by an experienced vascular neurologist (JGR in the MCSA cohort).

### Global and regional amyloid markers from amyloid-PET scans

The acquisition, preprocessing, and derivation of global Pittsburgh compound B (PiB)-PET standardized uptake value ratio (SUVR) in the MCSA population were described in Jack et al. [42] A global marker of amyloidosis was computed by averaging uptake across the prefrontal, orbitofrontal, parietal, temporal, anterior cingulate, and posterior cingulate/precuneus regions and normalizing this by uptake in cerebellar crus grey matter. In ADNI, amyloid PET was acquired using either florbetaben or florbetapir and the provided amyloid PET Centiloid values for the global composite region (frontal, anterior/posterior cingulate, lateral parietal, and lateral temporal cortices) normalized to whole cerebellum [43]. 

### Assessment of neuropathology and pathological SVD scales on postmortem tissue

**Fig. 2:**
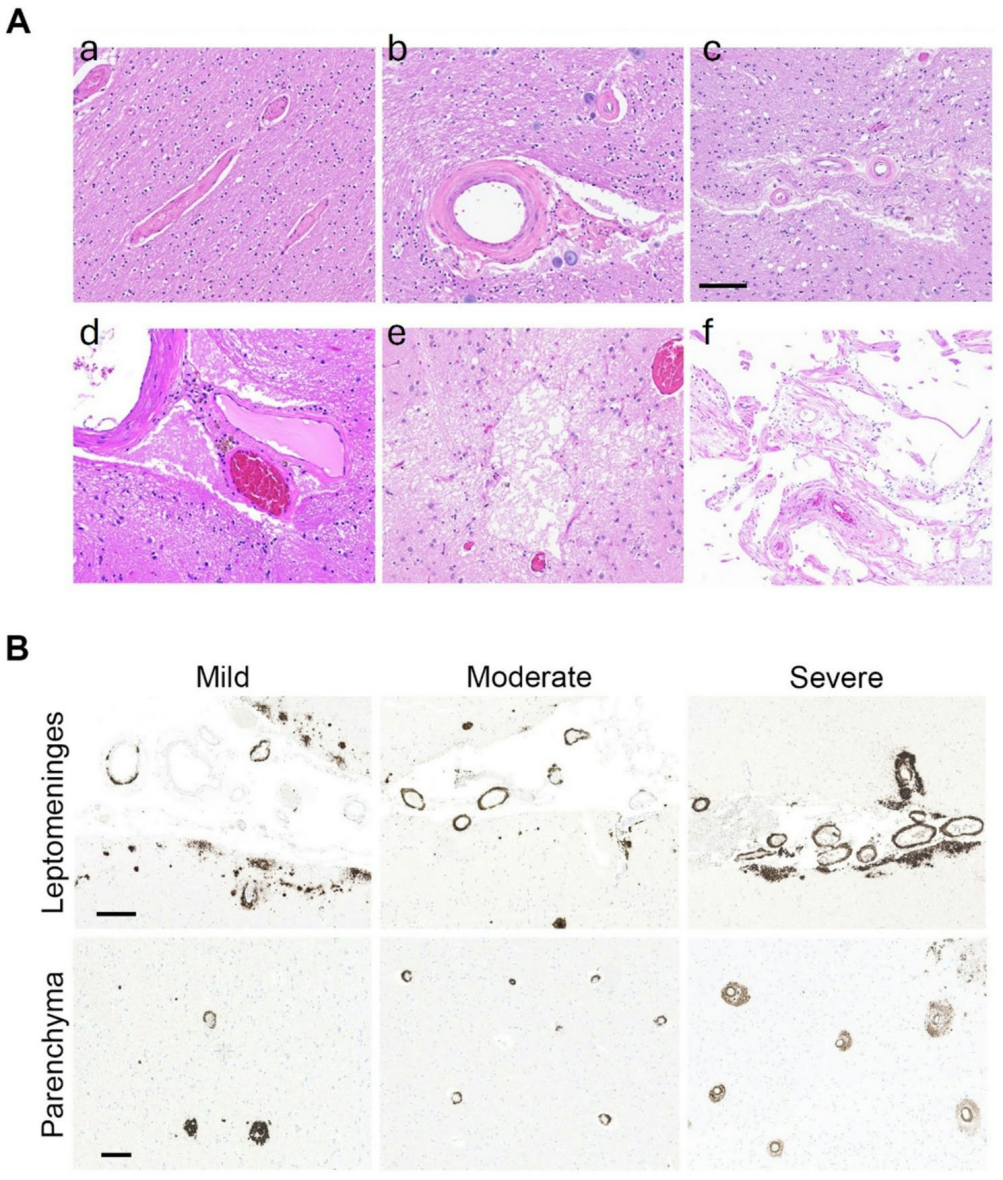
Neuropathologic sections from a representative sample. **A**) findings from Kalaria scale: **(a)** normal arteriole, **(b)** arteriolosclerosis, **(c)** perivascular rarefaction, **(d)** perivascular hemosiderin deposition, **(e)** microinfarct, and **(f)** subcortical infarct. **B)** sample immunostained with amyloid beta (Aβ) to illustrate cerebral amyloid angiopathy scores (scale of 0–3), reflecting increasing severity in leptomeningeal and parenchymal vessels. Mild (CAA score of 1) shows sparse positively stained vessels; moderate (CAA score of 2) shows Aβ deposition in several vessels with circumferential staining; severe (CAA score of 3) shows widespread circumferential vascular positivity. Scale bar: 100 μm; images: white-balanced

Autopsies were performed on a group of independent samples from the MCSA cohort with antemortem dMRI and postmortem SVD measures. Neuropathologic severity of SVD was retrospectively evaluated using modified Kalaria [23, 24] and CAA scales [44], based on hematoxylin and eosin stained sections, as published previously [20]. Briefly, the Kalaria scale incorporates scores from neocortical regions (0–6 points) and basal ganglia (0–4 points). This scale evaluates vessel wall pathology (arteriolosclerosis, CAA), perivascular and WM changes, cortical microinfarcts, and large cortical infarcts **(**Fig. 2A**)**. CAA burden was graded in the frontal, parietal, temporal, occipital, and hippocampal regions using the Love consensus criteria [44]. CAA severity in both cortical and parenchymal areas of each region was summarized into four levels (0 = absent; 1 = scant CAA; 2 = involvement of ≥ 2 arterioles, some exhibiting circumferential Aβ deposition; and 3 = widespread arteriolar Aβ deposition, with many arterioles showing circumferential involvement) **(**Fig. 2B**)**.

### Assessment of cognitive performance

All MCSA participants underwent a comprehensive neuropsychological test battery covering four cognitive domains such as attention, memory, language, and visuospatial skills [45]. Global cognition z-scores were calculated from the z-transformation of the average of the four cognitive domains. ADNI participants followed a different test battery as published in the ADNI website [46]. In this study, we focused on domain-specific z-scores of attention/executive dysfunction and memory as primary outcomes, based on evidence that SVD subtypes affect cognition differently: HA-SVD impacts attention/executive function, while CAA-SVD affects semantic memory. Global cognitive performances were compared using global z-scores from MCSA and Mini Mental State Examination (MMSE) [47] scores from ADNI.

### Statistical analysis

**Fig. 3:**
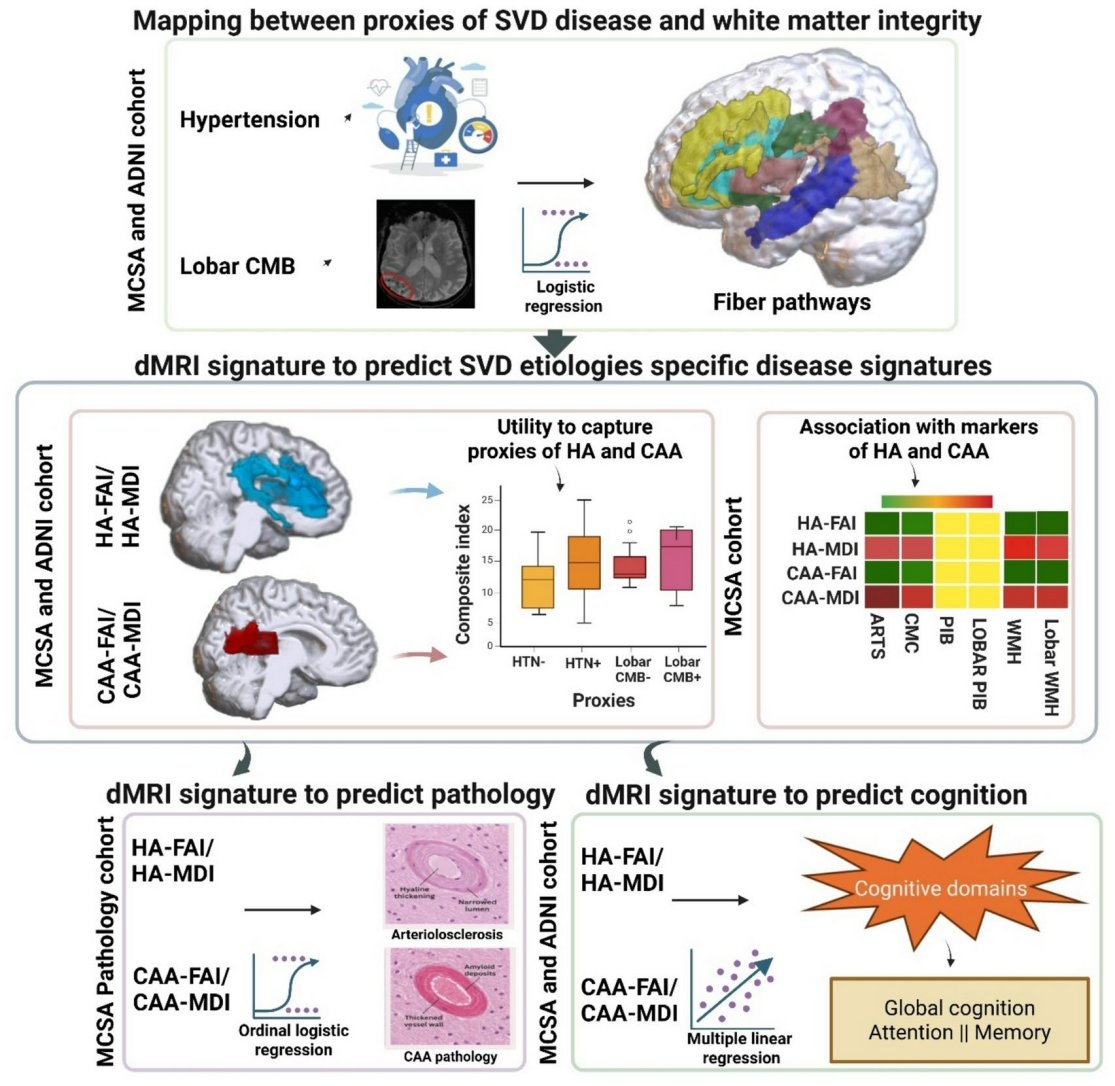
Overall statistical workflow. Top: Associations between proxies of hypertensive arteriopathy (HA; hypertension) and cerebral amyloid angiopathy (CAA; lobar microbleeds) with white-matter integrity were assessed using logistic regression across Mayo Clinic Study of Aging (MCSA) and Alzheimer’s Disease Neuroimaging Initiative (ADNI) cohorts. Middle: dMRI-derived HA and CAA signatures were generated and evaluated for their ability to capture SVD proxies and associate with HA/CAA markers. Bottom: These signatures were used to predict HA and CAA pathology in the MCSA pathology cohort and were related to cognitive performance (global, attention, memory) in MCSA and ADNI

The characteristics of the MCSA and ADNI participants are summarized with means and standard deviations for the continuous variables and counts and percentages for the categorical variables. PiB SUVR and WMH volumes were log transformed to reduce positive skew. The overall statistical workflow is shown in Fig. 3. The participants were assigned HTN status dichotomously based on a systolic blood pressure of ≥ 140 mm Hg or diastolic ≥ 90 mm Hg on 2 or more occasions or treatment for hypertension. The CMB positivity was also defined as binary (none, ≥ 1) and by location (any, deep, or lobar). Participants with co-occurring hypertension and lobar CMB status (6.9% in MCSA and 6.4% in ADNI) were retained and modeled accordingly, to improve transparency and avoid ambiguity regarding cohort composition.

### Association of regional dMRI markers with proxies of HA and CAA

Separate logistic regression models were fitted with regional dMRI measures (FA and MD) as predictors and hypertension or lobar CMB as outcomes, adjusting for age and sex. For all models, odds ratios and 95% confidence intervals (CIs) were obtained. Each dMRI measure was standardized into z-scores within each cohort prior to model fitting. To assess the etiological enrichment and robustness of the tract selection, we conducted sensitivity analyses in MCSA after excluding participants with mixed CMBs (1.2%). Although adjustments for education, other VRFs (diabetes, hyperlipidemia, and smoking), APOE ε4 carrier status, global amyloid burden or WMH may improve etiologic specificity, these variables lie on casual disease pathways and shares variance with microstructural integrity, hence were not included in the sensitivity analyses.

### Derivation of composite dMRI signatures

Composite dMRI indices (I) to serve as signatures of HA-SVD (HA-FAI, HA-MDI) and CAA-SVD (CAA-FAI, CAA-MDI) were computed using regional dMRI features associated with hypertension or lobar CMBs that survived Bonferroni correction for multiple comparisons (applied separately to FA and MD; 32 tests per metric, α = 0.05/32) in the MCSA cohort. For each composite, tract level FA or MD values from selected regions were combined using a voxel-weighted average, with weights reflecting each tract’s proportion of WM voxels within the selected set. This approach was chosen to obtain indices that capture diffusion abnormalities in proportion to their anatomical extent. The same tract sets derived in MCSA, which sampled participants from the general population and captures broad variability in vascular pathology, were applied to ADNI. We further compared the proxy groups (HTN- vs. HTN + for HA-SVD indices, lobar CMB- vs. CMB + for CAA-SVD indices) using boxplots and quantified the group differences using the Wilcoxon Rank Sum Test (p-value) and effect sizes Cohen’s d).

### Comparison of composite indices with standard global measures, and their utility in capturing aging and dementia markers

To compare the performance of composite dMRI signatures with established global SVD imaging measures (PSMD, FW, and WMH) in the MCSA cohort, we fit logistic regression models with each global or composite dMRI signatures as a predictor and SVD subtype proxies (HTN or lobar CMBs) as outcomes, adjusting for age and sex. Odds ratios and 95%CI were estimated for each predictor.

Further, the relationships between composite dMRI signatures and disease measures, including modified CMC score without hypertension (CMCm), WMH (global and lobar), and amyloid (global and lobar) burden, were assessed using Partial Pearson correlations controlling for age and sex.

### Validation of composite dMRI signatures using pathology

For confirmatory validation, we applied the MCSA-derived tract sets and voxel-weighting scheme to an independent and non-overlapping pathology dataset and derived the four composites. This biologically distinct, pathology-defined sample enabled internal-external validation and minimized concern about overfitting to the MCSA discovery cohort.

Associations between these dMRI signatures and postmortem pathology (Kalaria total score, Kalaria basal ganglia score, and CAA scores) were evaluated using weighted ordinal logistic regression models with adjustments for age at MRI, sex, and scan to death interval. Weights were calculated by 1/time from MRI scan to death which provided more weights for participants with scans closer to death. Composite dMRI signatures were standardized into z-scores prior to model fitting, and proportional odds ratios with 95% CI were estimated for each model. Sensitivity analyses were also performed in participants with scans within 5 years of death (*N* = 83, Supplementary Table 1). All analyses were conducted using polr() function from the MASS package in R.

### Association of composite dMRI signatures with cognitive performance

In the MCSA cohort, we assessed the associations between each composite dMRI signatures and cognitive outcomes (global cognition, attention, and memory z-scores) using multivariable linear regression models. Model 1 adjusted for age, sex, education, and cycle number (number of cognitive battery administration to adjust for practice effect). Model 2 further incorporated amyloid burden (Model 1 + log PIB). To understand the robustness of dMRI signatures to capture domain specificity above and beyond amyloid and WMH, we fit Model 3 (Model 2 + logWMH) with cognitive subdomains scores of attention and memory as outcomes and adjusted for multiple comparison correction using Bonferroni across cognitive subdomains and predictors. Each dMRI signature was entered separately as the primary predictor, and effect estimates are reported per one standard deviation increase in the corresponding signature. In the ADNI cohort, these models were repeated for cohort-specific cognitive outcomes, excluding cycle number because only baseline cognitive measurements available, and using amyloid-PET Centiloid as the amyloid imaging biomarker. All dMRI signatures were standardized into z-scores before model fitting, and model and partial R^2^ were obtained for each model.

## Results

The demographics, *APOE* ɛ4 status, intellectual enrichment variables, cognitive measures, and vascular and AD biomarker values of MCSA and ADNI participants are summarized in Table 1. The MCSA participants were younger than ADNI (mean [SD] age of MCSA participants was 66 [8.10] years and ADNI was 71 [7.15] years. Out of 1080 MCSA, 51% were males, 29% were *APOE ε4* positive, and mean education was 15 years. Among 549 ADNI participants, 45% were males, 40% were *APOE ε4* positive, and mean education was 16 years.

**Table 1:** Characteristics table of participants with the mean (SD) listed for the continuous variables and count (%) for the categorical variables

MCSA participants	All	HTN-	HTN+	LOBAR CMB-	LOBAR CMB+
N	1080	513	567	962	118
Age, yrs	66.31(8.99)	63.51 (8.19)	68.85 (8.93)	65.62 (8.51)	71.92 (10.72)
Male	555 (51%)	253 (49%)	302 (53%)	481 (50%)	74 (63)
*APOE* ɛ4	314 (29%)	156 (30%)	158 (28%)	274 (28%)	40 (34%)
Education, yrs	14.95 (2.51)	15.41 (2.35)	14.53 (2.57)	14.97 (2.48)	14.75 (2.71)
CMC	1.74(1.26)	0.80 (0.70)	2.59 (1.03)	1.67 (1.21)	2.26 (1.50)
PIB+	211 (20%)	71 (14%)	140 (25%)	164 (17%)	47 (40%)
PIB-PET, SUVR	1.46 (0.30)	1.41 (0.24)	1.51 (0.34)	1.45 (0.28)	1.61 (0.41)
PIB-PET, Centiloids	20.08 (26.57)	15.50 (21.36)	24.23 (29.95)	18.47 (24.59)	33.21 (36.76)
WMH	0.64 (0.79)	0.46 (0.59)	0.81 (0.91)	0.59 (0.71)	1.10 (1.20)
Global z-score	0.36 (1.02)	0.56 (0.95)	0.17 (1.05)	0.42 (0.98)	-0.10 (1.23)
Memory z-score	0.29 (1.07)	0.48 (0.99)	0.13 (1.12)	0.33 (1.06)	-0.03 (1.15)
Attention z-score	0.28 (1.00)	0.51 (0.91)	0.09 (1.03)	0.35 (0.95)	-0.24 (1.23)
Language z-score	0.16 (1.08)	0.32 (1.04)	0.02 (1.10)	0.21 (1.06)	-0.21 (1.17)
Visual-spatial z-score	0.32 (0.99)	0.44 (0.94)	0.21 (1.02)	0.35 (0.96)	0.04 (1.16)

ADNI participants	All	HTN-	HTN+	LOBAR CMB-	LOBAR CMB+
N	549	300	249	473	76
Age, yrs	71.09 (7.15)	69.87 (6.96)	72.56 (7.11)	70.70 (7.00)	73.49 (7.63)
Male	248 (45%)	119 (40%)	129 (52%)	213 (45%)	35 (46%)
*APOE* ɛ4	217 (40%)	128 (43%)	89 (36%)	184 (39%)	33 (43%)
Education, yrs	16.39 (2.33)	16.44 (2.24)	16.34 (2.44)	16.39 (2.28)	16.39 (2.63)
Amyloid-PET+	223 (41%)	123 (41%)	100 (40%)	185 (39%)	38 (50%)
Amyloid-PET, Centiloids	30.58 (42.73)	30.77 (41.94)	30.35 (43.74)	29.07 (41.66)	39.95 (48.08)
WMH	0.63 (0.70)	0.50 (0.51)	0.79 (0.85)	0.59 (0.60)	0.90 (1.12)
MMSE	27.96 (2.53)	28.31 (2.21)	27.53 (2.82)	28.06 (2.35)	27.33 (3.43)
Memory z-score	0.47 (0.73)	0.61 (0.73)	0.31 (0.71)	0.49 (0.71)	0.33 (0.87)
Executive function z-score	0.47 (0.68)	0.56 (0.62)	0.35 (0.72)	0.50 (0.62)	0.26 (0.92)

**Abbreviations**: HTN, hypertension; CMB, cerebral microbleed; APOE, apolipoprotein E; CMC, cardiovascular and metabolic conditions; PIB-PET, Pittsburg compound-B positron emission tomography; PIB +,  ≥ 25 centiloid; Amyloid PET+, -≥18 Centiloid; WMH, white matter hyperintensity volume as a % of TIV; MMSE, Mini-Mental State examination

Among the 147 autopsy cases, mean age at death was 84 [SD = 5.01] years and 52% were males with mean MRI scan time to death of 4.54 [SD = 2.75] years, 31% were *APOE ε4* carriers, and mean education was 15 years (Supplementary Table 1).

### Regional dMRI measures captured spatial heterogeneity associated with HA and CAA

**Fig. 4:**
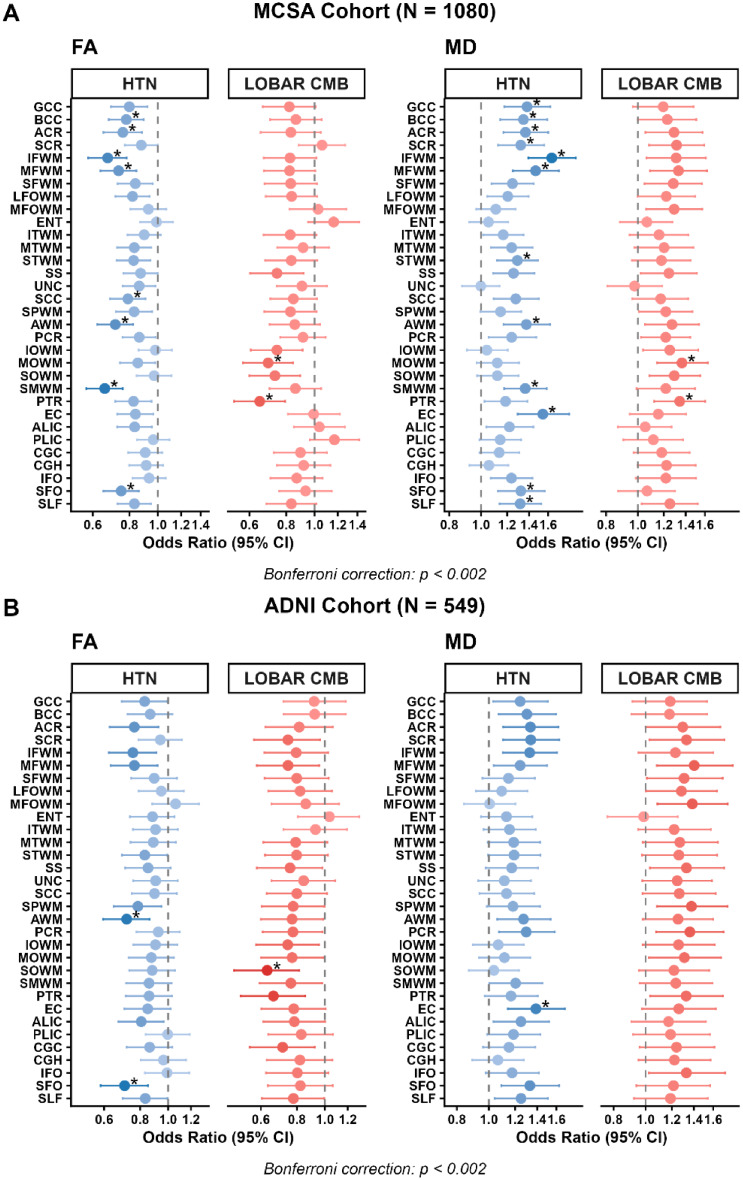
Association of dMRI signatures with proxies of Hypertensive arteriopathy (hypertension-[HTN]) and Cerebral amyloid angiopathy (lobar cerebral microbleeds [CMB]), adjusting for age and sex in the Mayo Clinic Study of Aging (MCSA) (**A**) and the Alzheimer’s Disease Neuroimaging Initiative (ADNI) (**B**). Higher FA = better integrity (OR < 1, lower risk); Higher MD = worse integrity (OR > 1, higher risk). FA, fractional anisotropy; MD, mean diffusivity. An asterisk (*) represents regions that survived Bonferroni correction and the x axis is log-scaled

Logistic regression analyses revealed distinct spatial patterns in the association between dMRI indices and proxies of HA and CAA after Bonferroni correction of *p* < 0.002. In the MCSA cohort, hypertension (a proxy for HA) was associated with reduced FA, primarily in fronto-parieto-projection pathways, including the body and splenium of the corpus callosum, anterior corona radiata, inferior and middle frontal WM, angular and supramarginal WM, and the superior fronto-occipital fasciculus **(**Fig. 4A, Supplementary Table 2). MD demonstrated a broader pattern, encompassing all FA-affected regions as well as the genu of corpus callosum, superior corona radiata, superior temporal WM, external capsule, and superior longitudinal fasciculus. On the other hand, lobar CMBs (a proxy for CAA) were associated with FA reductions and MD elevations in occipito-parietal regions, notably in the middle occipital WM and posterior thalamic radiation. When we performed sensitivity analyses after excluding participants with mixed CMBs, we found similar significant tract patterns though the lobar CMB-MD associations didn’t survive the Bonferroni threshold (Supplemental Fig. 1).

In the ADNI cohort, the associations were observed in fewer tracts **(**Fig. 4B, Supplementary Table 2) with attenuated effect sizes, but effect directions were fully concordant with MCSA (Supplemental Fig. 2). Similar to MCSA, hypertension was associated with reduced FA in angular WM and superior fronto-occipital fasciculus. For MD, only external capsule survived Bonferroni corrected significance for hypertension, compared with multiple lobar tracts in MCSA. However, lobar CMB showed significant associations with superior occipital WM.

**Fig. 5:**
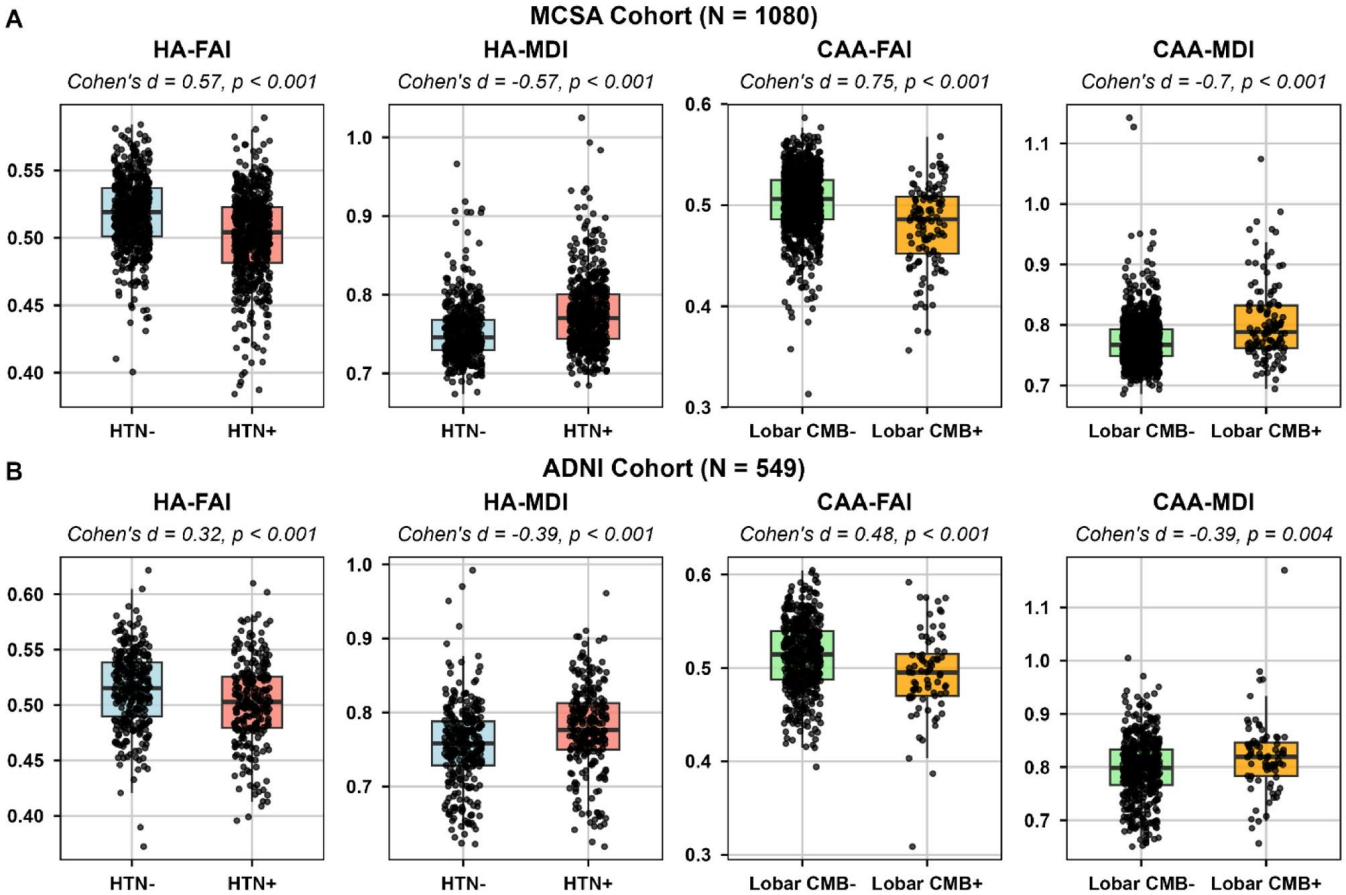
Comparison of dMRI signatures across proxies of hypertensive arteriopathy (HA) and cerebral amyloid angiopathy (CAA) in the Mayo Clinic Study of Aging (MCSA) (**A**) and Alzheimer’s Disease Neuroimaging Initiative (ADNI) (**B**). HA, hypertensive arteriopathy; CAA, cerebral amyloid angiopathy; FA, fractional anisotropy; MD, mean diffusivity; I, Index; HTN, hypertension; CMB, cerebral microbleeds

Notably, the derived composite regional dMRI indices were significantly differed between groups with and without HTN (*p* < 0.001) and lobar CMBs (*p* < 0.005) in both MCSA **(**Fig. 5A**)** and ADNI **(**Fig. 5B**)**.

### Differential association of composite dMRI indices and global SVD measures with SVD proxies

**Fig. 6:**
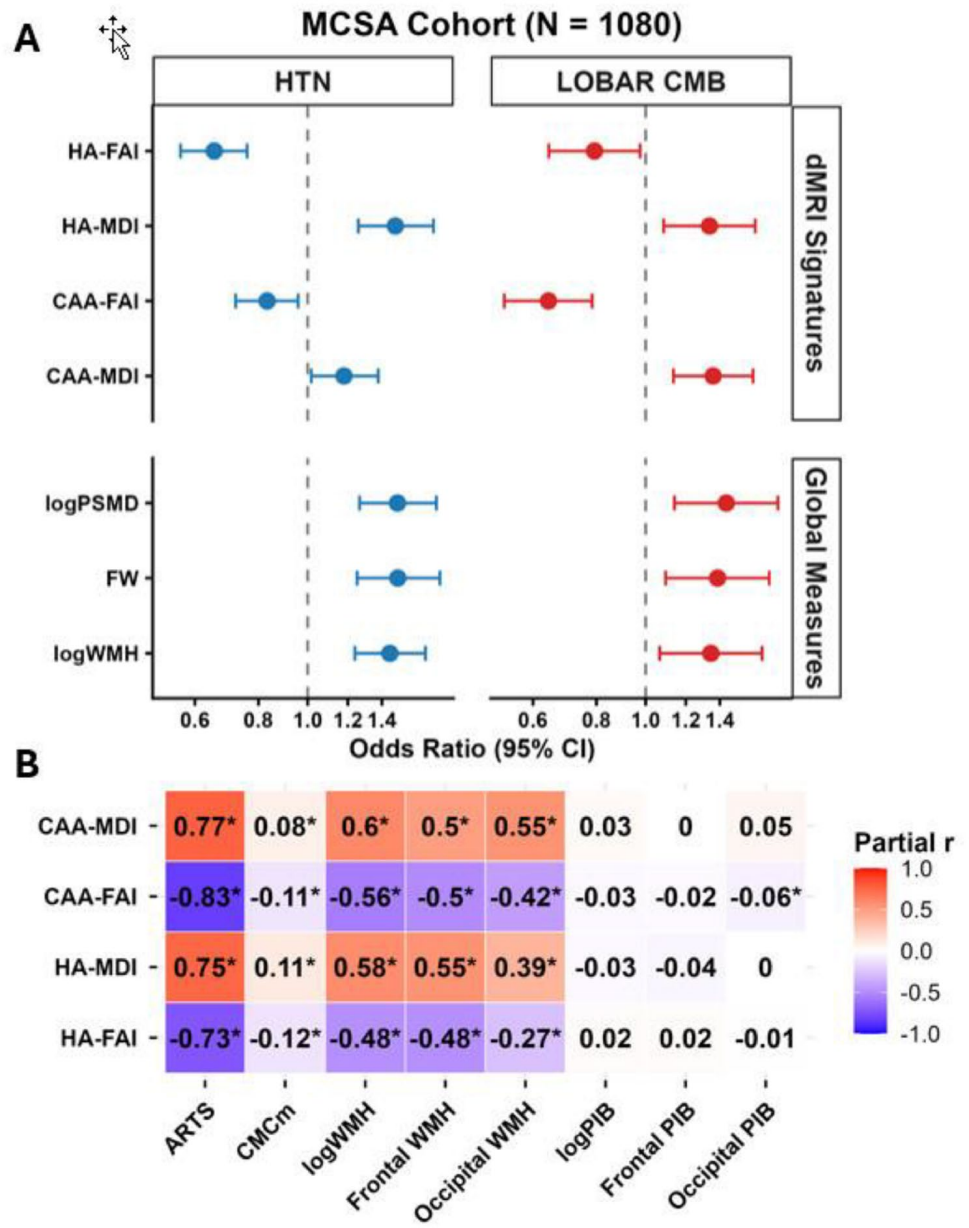
Association of global indices and composite dMRI signatures with proxies of hypertensive arteriopathy (hypertension-HTN) and cerebral amyloid angiopathy (lobar cerebral microbleeds [CMB]) (**A**) and association between dMRI signatures and established markers of HA and CAA in the community dwelling Mayo Clinic Study of Aging (MCSA) cohort with prevalent vascular disease (**B**). Higher FA = better integrity (OR < 1, lower risk); Higher MD = worse integrity (OR > 1, higher risk). HA, hypertensive arteriopathy; CAA, cerebral amyloid angiopathy; FA, fractional anisotropy; MD, mean diffusivity: I, Index; PSMD, peak-width skeletonized mean diffusivity; FW, free water; CMCm, cardiovascular and metabolic composite without hypertension; WMH, white matter hyperintensity % of TIV, PIB, Pittsburgh compound-B positron emission tomography; ARTS, arteriolosclerosis. In figure A, x axis is log scaled and in figure B, an asterisk (*) represents *p* < 0.05

All global measures were strongly associated with both hypertension and lobar CMBs in the MCSA cohort, highlighting their inability to distinguish etiologies **(**Fig. 6A**)**. In contrast, the regional composite dMRI signatures showed a slight stronger correlation to the proxy that was used to develop the dMRI indices as expected. Additionally, all dMRI indices were significantly associated with ARTS score, a neuroimaging marker of arteriolosclerosis, and CMCm, a measure of systemic vascular injury, after adjusting for age and sex **(**Fig. 6B**)**. Interestingly, CAA-SVD indices showed stronger associations with occipital WMH (partial *r* = -0.42 and 0.55, *p* < 0.001, respectively for CAA-FAI and CAA-MDI), a known marker of CAA, compared to HA-SVD indices (partial *r* = -0.27 and 0.39, *p* < 0.001, respectively for HA-FAI and HA-MDI). While all dMRI signatures were robustly associated with global WMH burden, none showed significant associations with global amyloid burden (*p* > 0.05). Notably, a weak association of CAA-FAI with occipital PIB was observed (partial *r* = -0.06, *p* < 0.05).

### Antemortem dMRI signatures captured pathological disease changes

Among the dMRI indices calculated within the pathology sample, HA-MDI showed a strong association with Kalaria total score (OR = 2.41, 95% CI: 1.33–4.36, *p* = 0.004) in the overall sample and in participants scanned within 5 years of death (OR = 2.41, 95% CI: 1.19–4.90, *p* = 0.015), after adjusting for age at MRI and time interval from scan to death (Table 2**)**. Similarly, HA-MDI was significantly associated with Kalaria basal ganglia score, which measures the vulnerability of basal ganglia due to hypertensive arteriolosclerosis. For CAA pathology, both CAA-FAI and CAA-MDI were associated with CAA pathological score across comparisons. Higher CAA-FAI values were associated with lower odds of severe CAA pathology (OR = 0.46, 95% CI: 0.27–0.80, *p* = 0.006 for all participants and OR = 0.35, 95% CI: 0.18–0.68, *p* = 0.002 within 5 years), whereas higher CAA-MDI values were associated with increased odds of severe pathology (OR = 2.08, 95% CI: 1.26–3.44, *p* = 0.004 and OR = 2.71, 95% CI: 1.47–4.98, *p* = 0.001 within 5 years) for all participants. These findings underscore the utility of dMRI signatures to capture subtype specific pathological severity.

**Table 2:** Weighted ordinal regression models for predicting pathology scores with an adjustment for age at MRI, sex, and time from scan to death and weighted for time. Higher FAI = better integrity (OR < 1 low risk); Higher MDI = worse integrity (OR > 1 high risk)

Outcome	Composite dMRI signature	All participants (*N* = 147)		Within 5 years (*N* = 83)	
		OR [95% CI]	*p*-value	OR [95% CI]	*p*-value
**Imaging signature predicting pathological Kalaria total score**					
	HA-FAI	0.86 [0.50–1.48]	0.590	0.97 [0.53–1.77]	0.913
	HA-MDI	2.41 [1.33–4.36]	**0.004**	2.41 [1.19–4.90]	**0.015**
	CAA-FAI	0.64 [0.36–1.13]	0.126	0.67 [0.35–1.30]	0.239
	CAA-MDI	1.41 [0.83–2.42]	0.205	1.36 [0.73–2.54]	0.335
**Imaging signature predicting pathological Kalaria basal ganglia score**					
	HA-FAI	1.05 [0.60–1.83]	0.876	1.28 [0.67–2.42]	0.451
	HA-MDI	2.44 [1.36–4.39]	**0.003**	2.29 [1.14–4.57]	**0.020**
	CAA-FAI	0.78 [0.46–1.33]	0.358	0.83 [0.45–1.52]	0.539
	CAA-MDI	0.92 [0.56–1.52]	0.752	0.84 [0.47–1.50]	0.544
**Imaging signature predicting pathological CAA score**					
	HA-FAI	0.67 [0.40–1.12]	0.130	0.57 [0.32–1.01]	0.055
	HA-MDI	1.12 [0.64–1.97]	0.693	1.49 [0.76–2.93]	0.241
	CAA-FAI	0.46 [0.27–0.80]	**0.006**	0.35 [0.18–0.68]	**0.002**
	CAA-MDI	2.08 [1.26–3.44]	**0.004**	2.71 [1.47–4.98]	**0.001**

**Abbreviations**: HA, hypertensive arteriopathy; CAA, cerebral amyloid angiopathy; FA, fractional anisotropy; MD, mean diffusivity; I, index

### dMRI signatures showed selective cognitive relevance to specific domains beyond AD and SVD markers

**Fig. 7:**
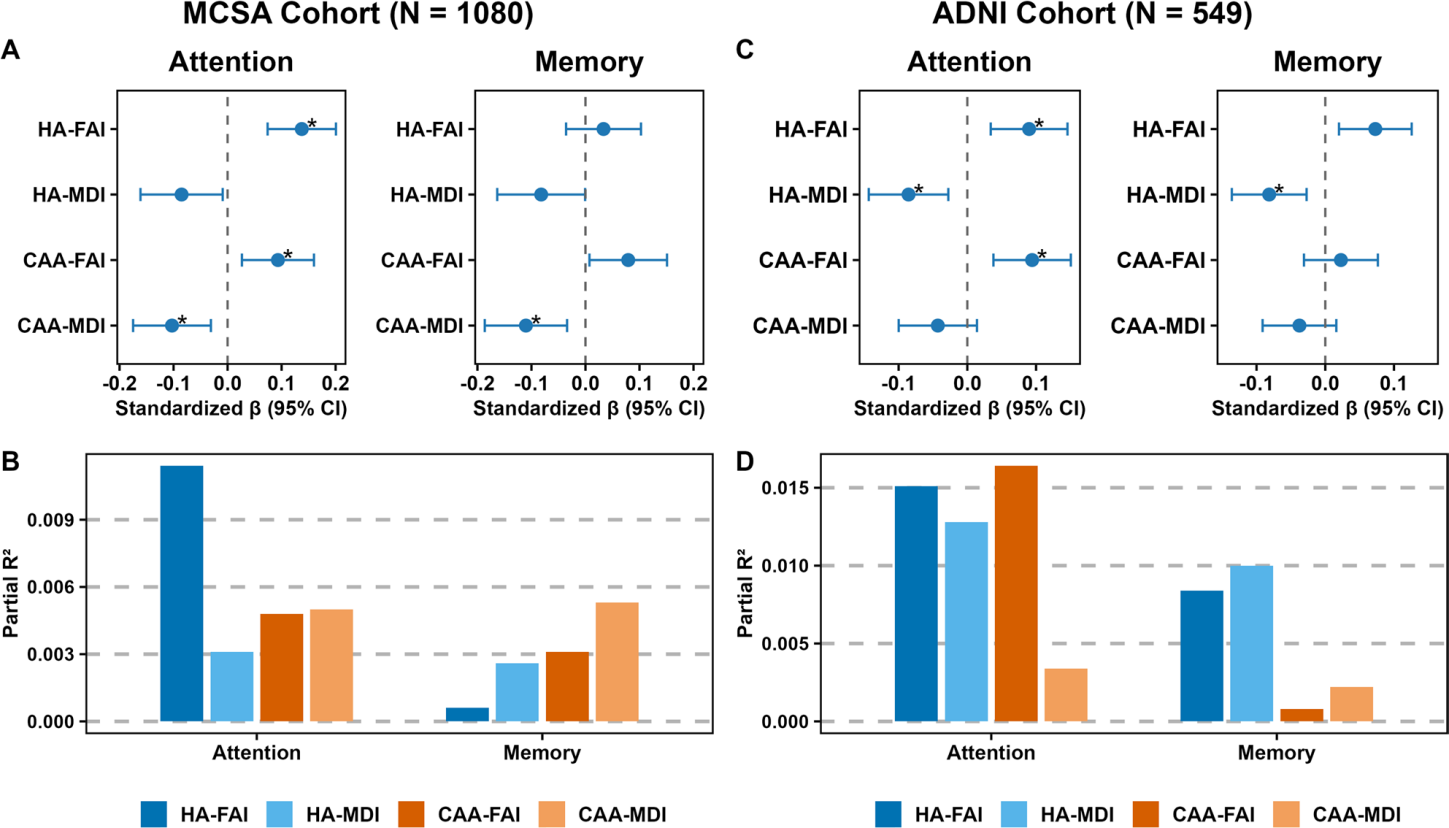
Association between composite dMRI signatures and cognitive subdomains in the Mayo Clinic Study of Aging (MCSA) (**A**) and Alzheimer’s Disease Neuroimaging Initiative (ADNI) (**B**) independent of amyloid and white matter hyperintensity. Model 3 (Cognition ~ age + sex + education + cycle number + composite index + Amyloid PET + WMH). ADNI models excluded cycle number as there were only baseline cognitive measurements. FA, fractional anisotropy; MD, mean diffusivity; HA, hypertensive arteriopathy; and CAA, cerebral amyloid angiopathy; and I, index, and MMSE, Mini-Mental state examination. An asterisk (*) represents associations that survived Bonferroni correction across domains and outcomes (*p* < 0.0125)

In MCSA, all dMRI signatures were associated with global cognition, attention, and memory z-scores in model 1 and model 2 (Supplemental Fig. 3A). In model 3 (Fig. 7A, Supplemental Table 2), all dMRI signatures remained significant predictors of global cognition and attention (*p* < 0.05), while HA-FAI index did not predict memory (β = 0.03, *p* = 0.345). Notably, in model 3 with subdomain scores, among the dMRI signatures, HA-FAI and CAA-SVD indices showed significant association with attention scores and HA-FAI explained the most variance (partial R² ≈ 0.011), whereas only CAA-MDI associated with memory and contributed most to the domain specificity (partial R² ≈ 0.01) after multiple comparison correction **(**Fig. 7B**).** These findings indicate that HA-related microstructural changes primarily affect attention, while CAA-related changes are more relevant for memory, even after accounting for amyloid and WMH. In ADNI, associations were less consistent and more sensitive to covariate adjustments (Supplemental Fig. 3B). While HA-SVD indices contributed more than CAA-SVD across domains (Fig. 7C&D), several associations, particularly CAA-MDI with attention as well as CAA-FAI and CAA-MDI with memory lost significance in Model 3.

## Discussion

In this study, we developed regional dMRI-derived signatures to characterize microangiopathic profiles of HA and CAA in two cohorts and validated them against disease-specific imaging markers, pathology, and cognition. The main findings were: (1) regional diffusion markers revealed distinct spatial patterns for HA (predominant fronto-parieto-projection) and CAA (occipito-parietal); (2) dMRI signatures captured disease-specific vascular injury patterns, with more stronger associations of CAA-SVD indices with occipital WMH and amyloid than HA-SVD indices; (3) Antemortem HA-SVD and (HA-MDI) indices were strongly associated with postmortem Kalaria total and basal ganglia scores across samples, whereas, CAA-SVD indices consistently predicted CAA pathology; and (4) In the MCSA cohort, all indices were associated with global cognition and subdomain scores beyond demographics and amyloid; notably HA-FAI was more strongly associated with attention, whereas CAA-MDI was significantly associated with memory after adjusting for amyloid, WMH, and multiple comparisons.

### Regional dMRI measures reveal spatial heterogeneity in SVD subtypes of HA and CAA

We found selective regional vulnerability of WM microstructure associated with distinct SVD profiles in MCSA and ADNI, though the power was limited in ADNI due to sampling design and cohort characteristics. HA-SVD proxy was associated with decreased FA and extensive MD elevations in fronto-temporo-parietal-subcortical regions, consistent with prior evidence that chronic hypertension and arteriosclerosis [48–50] impair long range WM tracts [51], including corpus callosum, corona radiata, anterior thalamic radiation, superior longitudinal fasciculus, inferior fronto-occipital fasciculus, and [52–54] inferior longitudinal fasciculus [55]. The observed broader MD changes suggest diffuse microstructural compromise due to both extracellular water accumulation and axonal degeneration [55], hallmark features of HA-SVD. The frontal predominance aligns with greater vulnerability of penetrating arteries from anterior circulation due to HA [38, 56–58]. 

On the other hand, CAA-SVD proxy was associated with occipito-parietal WM damage, specifically in middle occipital WM and posterior thalamic radiation, consistent with neuroimaging and histopathological evidence of posterior predilection of CAA-related pathology [59, 60] and amyloid deposition preferentially affecting posterior cortical and subcortical vessels, leading to regionally selective WM injury [61–63]. The spatial pattern of MD changes mirrored FA, reinforcing that CAA-related damage may be spatially confined yet detectable across diffusion metrics [19, 64]. Previous studies also report diffuse microstructural WM alterations with lobar CMBs, independent of age, sex, cardiovascular risk factors and other SVD imaging biomarkers [19, 64]. Liu et al. [19] reported localized destruction in the internal capsule and genu of corpus callosum, suggesting regional vulnerability due to neuroanatomical organization. Although the exact mechanisms remain unclear, it is plausible that CMBs may contribute to WM damage either through shared pathways, including APOEɛ4 carriership, cardiovascular risk factors, inflammation, or blood brain barrier dysfunction leading to local WM tissue injury [65, 66]. 

Importantly, the tract-level diffusion effect estimates showed moderate cross-cohort concordance between MCSA and ADNI. Additionally, effect sizes were systematically attenuated in ADNI, consistent with less pronounced regional patterns despite broadly preserved spatial organization (Supplemental Fig. 2). These findings indicate that ADNI provides partial external replication rather than full confirmation of the MCSA findings.

### Composite dMRI indices were associated with distinct disease specific signatures

Previous studies have advanced our understanding of SVD subtypes using composite imaging biomarkers such as total CAA-SVD score, which correlates with pathologic vasculopathic changes and symptomatic lobar intracerebral hemorrhage [10], as well as HA-SVD, CAA-SVD, and global SVD scores, which reflect disease specific severity and predict cognitive domains [9] and Alzheimer’s disease subtypes. However, these scores primarily capture established lesion markers, often after irreversible changes have happened in the brain. In contrast, the current study introduced novel FA-and MD-based composite dMRI signatures, which reflect early microstructural WM changes, often preceding visible lesions. We found significant differences in dMRI signatures between groups stratified by hypertension and lobar CMB status in both cohorts, further confirming their sensitivity to subclinical injury.

Importantly, our findings highlight the added value of composite regional dMRI signatures over global dMRI measures in characterizing the microstructural impact of distinct vascular pathologies. While the global diffusion measures significantly captured HA-and CAA-related alterations, supporting their role as indicators of overall disease burden, they showed limited ability to distinguish SVD subtypes compared with the composite dMRI signatures. In line with our findings, prior work from well-characterized memory clinic cohort at MGH reported comparable global PSMD values between probable CAA and arteriolosclerosis [18], but their regional approach revealed occipital-frontal PSMD gradients in CAA compared to non-SVD and SVD groups, suggesting that spatial diffusion patterns may better distinguish SVD phenotypes. Together, these findings emphasize the utility of regional dMRI metrics in disentangling the heterogeneous effects of HA and CAA on WM integrity.

Our results also provide insights into the relative performance of dMRI signatures in capturing regional cerebral vulnerabilities driven by distinct vascular mechanisms. For example, both dMRI signatures were significantly associated with a modified vascular composite independent of hypertension (CMCm) and ARTS, implicating its greater sensitivity to poorer WM health due to VRFs [28, 67, 68] such as diabetes [69, 70] and hyperlipidemia [68] as well as arteriolosclerosis. Consistent with the posterior predominant distribution of cerebral amyloid deposition in CAA, occipital WMH showed a stronger negative association with CAA-SVD indices than HA-SVD indices, independent of age and sex. This supports the notion of more localized posterior WM damage in association with CAA, as demonstrated in prior studies showing posterior-predominant WMH patterns linked to CAA [61–63]. Nevertheless, frontal WMH showed comparable associations with both HA-SVD and CAA-SVD indices, indicating that frontal WM damage may arise from overlapping vascular mechanisms. While CAA may cause focal vascular disruption, its downstream effects such as inflammation, impaired perivascular clearance, and disruption of functional connectivity can contribute to extensive damage, including parenchymal WMH and global cortical atrophy [71]. Although Aβ deposition is implicated in WM damage in both CAA and atherosclerosis [10, 72], we found a weak association of CAA indices with occipital amyloid. Our current findings reinforce earlier evidence that WMH rather than amyloid is the predominant driver of WM injury [38, 73] reflected by indices associated with HA-SVD and CAA-SVD proxies.

#### dMRI signatures map to SVD subtypes on postmortem tissue

Although neuroimaging findings are useful for visualizing the SVD-related abnormalities, imaging-pathological correlation studies are needed to bridge the gap between in vivo imaging and postmortem neuropathology [62, 63, 74]. More recently, we also showed a greater predictive power for antemortem dMRI in capturing early diffuse WM changes using neuropathologic SVD scales compared to traditional SVD imaging biomarkers (WMH, microbleeds, and infarcts), which suggested the impracticability to replicate lesion identification in imaging versus histopathology might cause this heterogeneity [20]. Charidimou et al. [63]., and others [75] have shown that small positive lesions on diffusion weighted imaging correlate with total MRI SVD scores in pathologically proven CAA, suggesting it as an additional marker of disease severity [63]. A recent multimodal study from 19 pathologically proven CAA cases also found that ex vivo WMHs were linked to cortical vascular amyloid-β, but not arteriolosclerosis [61]. Compared to normal appearing WM, posterior confluent and subcortical multispot WMHs showed increased activated microglia and clasmatodendrosis, indicating chronic neuroinflammation and astrocytic injury—supporting their use as pathologically and diagnostically relevant markers in Boston criteria v2.0. Recent work also highlighted the diagnostic utility of the posterior-to-anterior WMH ratio to distinguish CAA from HA [62]. However, to date, no studies have developed dMRI signatures that reliably correlate with underlying pathological features of CAA and HA.

In this study, an HA-SVD index (HA-MDI), but not CAA-SVD indices, were consistently associated with Kalaria total and Kalaria basal ganglia score across all samples, implicating the greater sensitivity of an HA-SVD signature to both overall SVD burden and vulnerability of basal ganglia due to hypertensive arteriolosclerosis and lipohyalinosis. These findings align with our prior work linking FA of GCC and CGH to microvascular pathology captured by Kalaria scale, suggesting that vacuolation in the CC reflects antemortem WM changes [20]. Notably, CAA-SVD indices were consistently associated with pathological CAA scores across all samples, indicating their potential as a sensitive marker of amyloid-related microstructural damage. Together, these findings highlight the specificity of dMRI signatures in capturing distinct SVD subtypes and underscore the need for targeted imaging biomarkers to disentangle the contributions of coexisting vascular pathologies. Future research should validate these composites in larger cohorts and assess their utility in distinguishing mixed pathology cases.

#### dMRI signatures predicted cognitive performance beyond traditional imaging biomarkers of AD and SVD

Both HA-SVD and CAA-SVD indices were independently associated with global cognitive performance and subdomain scores after adjusting for demographics in both MCSA and ADNI, consistent with prior findings linking hypertension and CMB-related WM microstructural associations with late-life cognitive function [52, 76, 77]. Hypertension-induced WM damage typically affects processing speed and executive function more than memory [52], reflecting disruption of long-range fibers such as superior longitudinal fasciculus, uncinate fasciculus, anterior thalamic radiation, inferior fronto-occipital fasciculus, and anterior corpus callosum. These effects may result from endothelial dysfunction, blood-brain barrier dysfunction, oxidative stress, and neuroinflammation [78, 79]. Although growing evidence supports an association between CMBs and cognitive dysfunction [80, 81], the underlying mechanism remains debated. Lobar CMBs have been linked to declines in executive and visuospatial dysfunction function, information processing, and memory [80, 82], and reduced WM integrity in posterior thalamic radiation and tapetum mediates the association between lobar CMBs and visuospatial dysfunction in Aβ + CAA patients, supporting the CMB-related WM damage and subsequent cognitive impairment [60]. However, other studies reported inconsistent findings, with some failing to show significant associations between lobar-CMBs related network disruption and cognitive impairment, possibly due to low prevalence or ethnic variability [76]. 

In our study, HA-SVD and CAA-SVD indices were associated with lower global cognition and attention z-scores across all models in both cohorts. However, only CAA-SVD indices showed robust association with memory domain after adjusting for global measures of amyloidosis and WMH in MCSA participants, whereas these associations became non-significant in ADNI. This suggests that HA-SVD indices, which primarily impact deep perforating arteries, contribute to frontal and subcortical WM damage and are more closely linked to deficits in executive function and processing speed in MCSA. The observed CAA-SVD indices association with memory beyond amyloid and WMH, likely reflects CAA’s predilection for cortical and leptomeningeal vessels, that are anatomically and functionally connected to medial temporal lobe structures critical for memory [75]. The differential associations in MCSA underscore the value of phenotype-specific dMRI signatures in capturing the cognitive impact of distinct SVD mechanisms. But in ADNI, these associations were inconsistent. HA-SVD indices were associated with both attention and memory even after adjusting other measures. These findings highlight the importance of incorporating composite dMRI markers to better understand and quantify cognitive impairment due to SVD subtype and suggest that CAA-related effects may be less independent in ADNI, potentially due to differences in recruitment criteria (individuals on the spectrum of Alzheimer’s disease with higher amyloid pathology along with exclusion of those with lower vascular burden), and cognitive assessment protocols.

#### Relevance of dMRI signatures for clinical and research studies

Early identification of HA-SVD and CAA-SVD is critical for both improving mechanistic understanding and clinical trial designs in VCID and AD. Trials like SPRINT-MIND, PRESERVE, ACCORD-MIND, and INFINITY have supported the role of blood pressure control in modifying non-amyloid SVD (WMH, cerebral blood flow, and microstructural WM integrity) and associated cognitive/clinical decline [83–85]. Furthermore, CAA has emerged as key contributor to ARIA, a major adverse effect of anti-Aβ immunotherapies, underscoring the need for careful participant selection in anti-amyloid trials [86]. Ongoing trials for CAA require participants to meet the Boston Version 2.0 criteria for CAA, but intervening earlier may improve efficacy.

While inflammation, as well as hypoxia/hypoperfusion induced BBB degradation, have been proposed in SVD-related WM damage, the lack of understanding of the mechanistic pathways underlying pathophysiological processes limit the treatment options [87]. More recently, the Framework for Clinical trials in SVD (FINESSE) proposed DTI as one of the surrogate end points to assess treatment efficacy in VCID phase 2 trials [88]. Additionally, recent trials [15, 89] and initiatives, including MarkVCID consortium [90], have emphasized the utility of global dMRI markers for detecting early-stage microstructural changes and predicting dementia risk. However, aging and SVD-related damage often affect areas disproportionately across the brain. Derived from standard diffusion MRI sequences, these signatures are easily accessible, scalable, and reproducible across imaging sites and vendors, making them well-suited for multi-center trials and longitudinal studies, where consistency and sensitivity are critical. By complementing existing HA-SVD and CAA-SVD scores (which are based on lesion markers), the proposed composite indices may provide a more nuanced signal of early network disruption and later structural damage for individual in SVD risk stratification. These measures are intended to capture continuous variation across overlapping vascular processes rather than to replace established markers. Future integration of these etiology specific markers into VCID and AD trials remain exploratory and will require further validation, particularly in populations with mixed vascular and neurodegenerative pathology.

### Strengths and limitations

The key strengths of this study include a large community-based sample with imaging biomarkers of SVD and AD, detailed vascular profiles, and comprehensive neuropsychological assessments, along with validation of composite dMRI signatures in an independent, well-characterized pathology dataset. The limitations include cross-sectional design, the focus on traditional dMRI markers rather than regional or lobar PSMD values [18, 91], and CMB assessments with T2* MRI instead of more sensitive susceptibility weighted imaging. Although we used a consistent logistic regression approach across SVD proxies, the excess of zeros in lobar CMB may reduce statistical power and stability of estimates; also suggests that Hurdle or zero inflated model might be more appropriate in future work. Bonferroni correction was chosen over false discovery rate to enable strict feature selection for reproducible composites; however given the strong spatial correlation across tracts, we acknowledge that this conservative choice may introduce some ranking instability. Given differing outcome prevalence, any odds-based associations should be interpreted cautiously. We did not adjust for *APOE* ɛ4 or education in the primary models because *APOE* ɛ4 has not shown meaningful associations with vascular pathways in prior MCSA studies, and education exerts broad upstream effects on diffusion measures; including these variables would also introduce substantial multicollinearity in the models. While inclusion of other SVD imaging markers such as lacunes, infarctions, perfusion, and perivascular spaces maybe beneficial, dMRI markers are likely to capture overall damage due to lesions. Validation in ADNI supports the generalizability of the measurement to participants who enter AD clinical trials, though ADNI’s exclusion of individuals with elevated Hachinski Ischemic score reduces the frequency of SVD.

## Conclusion

In summary, hypertension and lobar CMBs-related WM microstructural damage are proxies of mechanistically distinct pathological SVD changes. While composite SVD scores remain essential for clinical staging and outcome prediction, dMRI signatures can provide a complementary and potentially sensitive tool for early detection and subtype differentiation in SVD in clinical and research settings. This study lays a foundation for developing highly sensitive composite metrics for SVD etiologies that can incorporate early SVD-related changes and better mechanistic understanding of disease progression. Future work will focus on using advanced dMRI techniques such as neurite orientation dispersion and density imaging, their comparison with HA-SVD and CA-SVD lesion scores, validation in diverse cohorts, and explore their utility in clinical decision-making.

## Supplementary Information

Below is the link to the electronic supplementary material.

Supplementary Material 1

## Data Availability

All the imaging datasets for MCSA are available for download from GAAIN.org. ADNI data are available for download from (https://adni.loni.usc.edu).
