# Supplementary material for: Regional diffusion imaging measures to disentangle SVD-related hypertensive arteriopathy versus cerebral amyloid angiopathy
Source: Mol Neurodegener. 2026 Apr 13;21:27. doi: 10.1186/s13024-026-00942-4 (PMC13185335; doi:10.1186/s13024-026-00942-4)

**ONLINE SUPPLEMENTARY MATERIALS**

**Regional Diffusion Imaging Measures to Disentangle SVD-related Hypertensive Arteriopathy versus Cerebral Amyloid Angiopathy**

### Sheelakumari Raghavan^1^, Scott A. Przybelski^2^, Robel K. Gebre^1^, Audrey Low^1^, Mingzhao Hu^2^, Robert. I. Reid^3^, [B. Gwen Windham](https://alz-journals.onlinelibrary.wiley.com/authored-by/Windham/B.+Gwen)^4^, [Heather J. Wiste](https://alz-journals.onlinelibrary.wiley.com/authored-by/Wiste/Heather+J.)^2^, Angela J. Fought^2^, Michael G. Kamykowski^3^, Aivi T. Nguyen^5^, Melissa E. Murray^6^, Val J. Lowe^1^, Clifford R. Jack Jr^1^, Ronald C. Petersen^7^, Jonathan Graff-Radford^7^, Prashanthi Vemuri^1^, for the Alzheimer’s Disease Neuroimaging Initiative*

^1^Department of Radiology, Mayo Clinic, Rochester, MN, 55905, USA

^2^ Department of Quantitative Health Sciences, Mayo Clinic, Rochester, MN, 55905, USA

^3^ Department of Information Technology, Mayo Clinic, Rochester, MN, 55905, USA

^4^Department of Medicine, The MIND Center, University of Mississippi Medical Center, Jackson, 39216, USA

^5^Department of Laboratory Medicine and Pathology, Mayo Clinic, Rochester, MN, 55905, USA

^6^Department of Laboratory Medicine and Pathology, Mayo Clinic, Jacksonville, Florida, 3224, USA

^7^ Department of Neurology, Mayo Clinic Rochester, MN, 55905, USA

*Data used in preparation of this article were obtained from the Alzheimer’s Disease Neuroimaging Initiative (ADNI) database (adni.loni.usc.edu). As such, the investigators within the ADNI contributed to the design and implementation of ADNI and/or provided data but did not participate in analysis or writing of this report. A complete listing of ADNI investigators can be found at: http://adni.loni.usc.edu/wp-content/uploads/how_to_apply/ADNI_Acknowledgement_List.pdf

**Corresponding Authors:** Sheelakumari Raghavan, PhD

Department of Radiology, Mayo Clinic

200 First Street SW, Rochester, MN 55905

Phone: (507)398-1288

Email: Raghavan.Sheela@mayo.edu

Prashanthi Vemuri, PhD

Department of Radiology, Mayo Clinic

200 First Street SW, Rochester, MN 55905

Phone: (507)538-0761

Fax:+1 507 284 9778

Email: [Vemuri.prashanthi@mayo.edu](mailto:Vemuri.prashanthi@mayo.edu)

**Supplemental Table 1:** Characteristics of autopsy participants with mean (SD) listed for continuous variables and count (%) for the categorical variables.

| **Characteristics** | All  N = 147 | < 5 years  N = 83 |
| --- | --- | --- |
| Age, yrs | 84.05 (5.01) | 84.41 (5.46) |
| Male, no. (%) | 76 (52%) | 52 (63%) |
| Education, yrs | 14.64 (3.10) | 14.65 (3.10) |
| Time to death, yrs | 4.54 (2.75) | 2.46 (1.36) |
| APOE ɛ4, no. (%) | 45 ( 31%) | 23 (28%) |
| Kalaria total score | 4.57 (2.38) | 4.39 (2.39) |
| Kalaria basal ganglia score | 1.54 (1.05) | 1.42 (1.03) |
| CAA score | 0.84 (0.87) | 0.92 (0.88) |

**Abbreviations:** APOE, apolipoprotein E; CAA, cerebral amyloid angiopathy.

**Supplemental Table 2**: **Logistic regression models evaluating the associations of dMRI signatures with proxies of hypertensive arteriopathy (hypertension-[HTN]) and Cerebral amyloid angiopathy (lobar cerebral microbleeds [CMB]), adjusting for age and sex in the Mayo Clinic Study of Aging (MCSA) and the Alzheimer’s Disease Neuroimaging Initiative (ADNI)**.

|  | **HTN-FA** | | **LOBAR-FA** | | **HTN-MD** | | **LOBAR-MD** | |
| --- | --- | --- | --- | --- | --- | --- | --- | --- |
|  | **MCSA** | **ADNI** | **MCSA** | **ADNI** | **MCSA** | **ADNI** | **MCSA** | **ADNI** |
| Tracts |  |  |  |  |  |  |  |  |
| GCC | 0.80 (0.69–0.92), p=0.002 | 0.83 (0.69–0.99), p=0.043 | 0.82(0.67–1.01), p=0.06 | 0.92 (0.72–0.1.18), p=0.499 | 1.38 (1.18–1.62), p=<0.001 | 1.24 (1.03 -1.51), p=0.025 | 1.19 (0.97–1.48), p=0.101 | 1.19 (0.91–1.53), p=0.197 |
| BCC | 0.78 (0.68–0.89), p<0.001 | 0.87 (0.72–1.03), p=0.116 | 0.86(0.70–1.06), p=0.154 | 0.92 (0.72–0.1.18), p=518 | 1.35 (1.14–1.59), p=<0.001 | 1.30 (1.07–1.59), p=0.009 | 1.23 (1.00–1.51), p=0.048 | 1.18 (0.91–1.53), p=0.217 |
| ACR | 0.76 (0.65–0.88), p<0.001 | 0.76 (0.63–0.93), p=0.007 | 0.83(0.65–1.05), p=0.122 | 0.81 (0.62–1.07), p=134 | 1.37 (1.17–1.60), p<0.001 | 1.33 (1.10–1.62), p=0.003 | 1.29(1.05–1.57), p=0.013 | 1.29 (1.00–1.68), p=0.052 |
| SCR | 0.88 (0.77–1.00), p=0.048 | 0.94 (0.79–1.12), p=0.482 | 1.06 (0.88–1.27), p=0.531 | 0.74 (0.57–0.96), p=0.028 | 1.32 (1.13–1.56), p<0.001 | 1.34 (1.10–1.63), p=0.003 | 1.31(1.08–1.59), p=0.006 | 1.33 (1.03–1.73), p=0.032 |
| IFWM | 0.67 (0.58–0.78), p<0.001 | 0.75 (0.62–0.91), p=0.004 | 0.82(0.67–1.01), p=0.066 | 0.80 (0.62–1.03), p=0.077 | 1.64(1.39–1.94), p<0.001 | 1.33 (1.10–1.61), p=0.003 | 1.31 (1.06–1.61), p=0.011 | 1.23 (0.95–1.60), p=0.119 |
| MFWM | 0.73 (0.64–0.85), p<0.001 | 0.76 (0.63–0.92), p=0.005 | 0.82 (0.67–1.01), p=0.056 | 0.75 (0.58–0.96), p=0.02 | 1.47 (1.25–1.73), p<0.001 | 1.24 (1.03–1.50), p=0.022 | 1.33 (1.09–1.62), p=0.005 | 1.40 (1.08–1.83), p=0.011 |
| SFWM | 0.84 (0.73–0.96), p=0.012 | 0.89 (0.74–1.07), p=0.234 | 0.83 (067–1.02), p= 0.074 | 0.80 (0.62–1.03), p=0.085 | 1.25 (1.08–1.45), p=0.003 | 1.15 (0.96–1.38), p=0.144 | 1.28 (1.05–1.57), p=0.015 | 1.31 (1.01–1.71), p=0.046 |
| LFOWM | 0.82 (0.72–0.94), p=0.004 | 0.95 (0.79–1.13), p=0.55 | 0.84 (0.68–1.02), p=0.08 | 0.82 (0.64–1.06), p=0.126 | 1.21 (1.04–1.39), p=0.011 | 1.09 (0.91–1.31), p=0.332 | 1.22 (1.00–1.49), p=0.053 | 1.28 (1.00–1.64), p=0.048 |
| MFOWM | 0.93 (0.81–1.07), p=0.302 | 1.06 (0.88–1.28), p=0.528 | 1.03 (0.82–1.29), p=0.813 | 0.86 (0.65–1.12), p=0.266 | 1.11 (0.97–1.27), p=0.141 | 1.00 (0.84–1.20), p=0.958 | 1.29 (1.06–1.57), p=0.011 | 1.38 (1.08–1.77), p=0.009 |
| ENT | 0.99 (0.87–1.12), p=0.868 | 0.88 (0.74–1.05), p=0.169 | 1.16(0.95–1.42), p=0.143 | 1.04 (0.81–1.32), p=0.772 | 1.05 (0.92–1.21), p=0.458 | 1.13 (0.95–1. 35), p=0.179 | 1.07 (0.88–1.27), p=0.50 | 0.99 (0.77–1.25), p=0.912 |
| ITWM | 0.90 (0.79–1.02), p=0.106 | 0.90 (0.75–1.08), p=0.271 | 0.83(0.67–1.02), p=0.07 | 0.93 (0.72–1.19), p=0.563 | 1.17 (1.01–1.35), p=0.031 | 1.15 (0.96–1.39), p=0.122 | 1.16(0.94–1.42), p=0.157 | 1.22 (0.95–1.58), p=0.123 |
| MTWM | 0.83 (0.73–0.95), p=0.007 | 0.89 (0.74–1.06), p=0.197 | 0.91 (0.75–1.12), p=0.383 | 0.79 (0.61–1.02), p=0.073 | 1.24 (1.07–1.44), p=0.004 | 1.19 (0.99–1.43), p=0.063 | 1.20 (0.98–1.48), p=0.081 | 1.27 (0.98–1.65), p=0.073 |
| STWM | 0.83 (0.72–0.94), p=0.005 | 0.83 (0.69–0.99), p=0.044 | 0.83 (0.68–1.02), p=0.08 | 0.80 (0.62-1.03), p= 0.078 | 1.29 (1.12–1.49), p<0.001 | 1.19 (0.99–1.43), p=0.059 | 1.18 (0.96–1.45), p=0.115 | 1.26 (0.97–1.64), p=0.083 |
| SS | 0.87 (0.76–1.00), p=0.048 | 0.85 (0.71–1.02), p=0.083 | 0.74 (0.60–0.92), p=0.006 | 0.76 (0.58–0.98), p=0.037 | 1.26 (1.09–1.45), p=0.002 | 1.17 (0.98–1.41), p=0.088 | 1.24 (1.02–1.51), p=0.03 | 1.33 (1.03–1.73), p=0.031 |
| UNC | 0.86 (0.76–0.98), p=0.025 | 0.90 (0.76–1.08), p=0.27 | 0.91 (0.74–1.10), p=0.324 | 0.84 (0.65-1.09), p=0.191 | 1.00 (0.88–1.14), p=0.972 | 1.11 (0.93–1.34), p=0.249 | 0.98 (0.81–1.19), p=0.813 | 1.25 (0.98–1.59), p=0.074 |
| SCC | 0.79 (0.69–0.91), p<0.001 | 0.90 (0.75–1.07), p=0.237 | 0.85(0.71–1.02), p=0.074 | 0.80 (0.63–1.02), p=0.068 | 1.28 (1.09–1.50), p=0.003 | 1.13 (0.94–1.37), p=0.204 | 1.17(0.96–1.42), p=0.108 | 1.26 (0.98–1.63), p=0.07 |
| SPWM | 0.83 (0.72–0.96), p=0.01 | 0.79 (0.65–0.95), p=0.013 | 0.83 (0.67–1.02), p=0.07 | 0.78 (0.60–1.00), p=0.047 | 1.15 (0.99–1.33), p=0.062 | 1.18 (0.99–1.43), p=0.075 | 1.22 (1.01–1.47), p=0.04 | 1.38 (1.08–1.77), p=0.01 |
| AWM | 0.72 (0.62–0.82), p<0.001 | 0.72 (0.60–0.86), p<0.001 | 0.86 (0.70–1.04), p=0.123 | 0.77 (0.60–0.99), p=0.039 | 1.37 (1.17–1.62), p <0.001 | 1.27 (1.06–1.54), p=0.011 | 1.27 (1.05–1.54), p=0.013 | 1.25 (0.98–1.60), p=0.069 |
| PCR | 0.86 (0.76–0.98), p=0.027 | 0.92 (0.78–1.10), p=0.371 | 0.91 (0.76–1.09), p=0.317 | 0.77 (0.61–0.98), p=0.036 | 1.24 (1.05–1.47), p=0.012 | 1.30 (1.07–1.58), p=0.009 | 1.22 (1.02–1.44), p=0.026 | 1.36(1.08–1.72), p=0.01 |
| IOWM | 0.98 (0.86–1.12), p=0.765 | 0.90 (0.76–1.08), p=0.258 | 0.74(0.61–0.91), p=0.004 | 0.74 (0.58–0.95), p=0.02 | 1.04 (0.91–1.21), p=0.587 | 1.07 (0.89–1.27), p=0.473 | 1.25 (1.04–1.53), p=0.024 | 1.26 (0.98–1.62), p=0.072 |
| MOWM | 0.85 (0.74–0.98), p=0.025 | 0.87 (0.73–1.05), p=0.146 | 0.69(0.57–0.85), p<0.001 | 0.77 (0.60–0.99), p=0.039 | 1.12 (0.97–1.31), p=0.136 | 1.11 (0.93–1.34), p=0.234 | 1.36 (1.14–1.63), p<0.001 | 1.31 (1.02–1.69), p=0.034 |
| SOWM | 0.97 (0.85–1.11), p=0.677 | 0.88 (0.73–1.06), p=0.172 | 0.73 (0.60–0.89), p=0.002 | 0.63 (0.48–0.82), p<0.001 | 1.12 (0.97–1.30), p=0.126 | 1.03 (0.87–1.23), p=0.701 | 1.29 (1.08–1.55), p=0.005 | 1.22 (0.95–1.56), p=0.114 |
| SMWM | 0.66 (0.57–0.76), p<0.001 | 0.86 (0.72–1.03), p=0.1 | 0.86 (0.70–1.05), p=0.1444 | 0.76 (0.59–0.98), p=0.033 | 1.36 (1.18–1.58), p<0.001 | 1.20 (1.00–1.45), p=0.047 | 1.22 (0.99–1.49), p=0.06 | 1.23 (0.96–1.56), p=0.103 |
| PTR | 0.83 (0.72–0.95), p=0.008 | 0.86 (0.71–1.03), p=0.105 | 0.65 (0.53–0.79), p<0.001 | 0.66 (0.51–0.85), p=0.002 | 1.19 (1.02–1.38), p=0.026 | 1.17 (0.97–1.41), p=0.103 | 1.34 (1.12–1.60), p=0.001 | 1.33 (1.03–1.71), p=0.028 |
| EC | 0.84 (0.73–0.96), p=0.015 | 0.85 (0.71–1.02), p=0.082 | 0.99 (0.81–1.22), p=0.945 | 0.78 (0.60–1.01), p=0.058 | 1.54 (1.29–1.86), p<0.001 | 1.39 (1.14–1.70), p=0.001 | 1.15 (0.94–1.40), p=0.155 | 1.26 (0.97–1.63), p=0.078 |
| ALIC | 0.83 (0.73–0.95), p=0.009 | 0.81 (0.67–0.96), p=0.02 | 1.04 (0.85–1.27), p=0.718 | 0.78 (0.61–1.002), p=0.051 | 1.22 (1.04–1.44), p=0.018 | 1.25 (1.03–1.52), p=0.024 | 1.05 (0.87–1.27), p=0.588 | 1.17 (0.91–1.52), p=0.228 |
| PLIC | 0.96 (0.85–1.10), p=0.578 | 1.00 (0.84–1.19), p=0.971 | 1.17 (0.96–1.42), p=0.116 | 0.83 (0.64–1.07), p=0.151 | 1.14 (0.99–1.32), p=0.073 | 1.19 (0.99–1.43), p=0.07 | 1.11 (0.90–1.37), p=0.309 | 1.19 (0.92–1.56), p=0.203 |
| CGC | 0.91 (0.79–1.03), p=0.144 | 0.86 (0.72–1.03), p=0.11 | 0.90 (0.73–1.10), p=0.294 | 0.71 (0.55–0.92), p=0.011 | 1.14 (0.99–1.31), p=0.074 | 1.15 (0.96–1.38), p=0.132 | 1.18 (0.97–1.44), p=0.094 | 1.24 (0.96–1.61), p=0.105 |
| CGH | 0.91 (0.80–1.04), p=0.18 | 0.96 (0.80–1.15), p=0.685 | 0.92 (0.74–1.13), p=0.423 | 0.82 (0.63–1.06), p=0.136 | 1.06 (0.92–1.21), p=0.432 | 1.06 (0.89–1.27), p=0.494 | 1.22 (1.00–1.50), p=0.054 | 1.22 (0.95–1.57), p=0.117 |
| IFO | 0.93 (0.82–1.06), p=0.301 | 0.99 (0.83–1.18), p=0.925 | 0.87(0.71–1.07), p=0.177 | 0.80 (0.63–1.03), p=0.083 | 1.24 (1.07–1.43), p=0.004 | 1.17 (0.98–1.42), p=0.087 | 1.22 (0.98–1.50), p=0.069 | 1.33 (1.02–1.74), p=0.036 |
| SFO | 0.75 (0.65–0.86), p<0.001 | 0.71 (0.58–0.85), p<0.001 | 0.93 (0.76–1.14), p=0.499 | 0.82 (0.63–1.07), p=0.138 | 1.32 (1.13–1.56), p<0.001 | 1.33 (1.09–1.63), p=0.005 | 1.07 (0.87–1.30), p=0.526 | 1.21 (0.94–1.57), p=0.138 |
| SLF | 0.83 (0.73–0.95), p=0.006 | 0.83 (0.70–0.99), p=0.044 | 0.83(0.68–1.01), p=0.068 | 0.78 (0.60–1.00), p=0.047 | 1.32 (1.14–1.53), p<0.001 | 1.25 (1.04–1.51), p=0.018 | 1.25 (1.03–1.52), p=0.025 | 1.19 (0.92–1.54), p=0.187 |
| Genu, body, and splenium of corpus callosum (GCC, BCC, SCC); inferior, middle and superior frontal white matter (IFWM, MFWM, SFWM); lateral and middle orbito frontal WM (LFOWM and MFOWM); uncinate fasciculus (UNC); superior fronto-occipital fasciculus (SFO); inferior fronto-occipital fasciculus (IFO); superior longitudinal fasciculus(SLF); cingulum (CGC); parahippocampal cingulum (CGH); anterior, superior, and posterior corona radiata (ACR, SCR, PCR); inferior, middle, and superior temporal WM (ITWM, MTWM, STWM); entorhinal WM (ENT); sagittal stratum (SS); external capsule (EC); anterior and posterior limb of internal capsule(ALIC and PLIC); superior parietal WM (SPWM); angular WM (AWM); supramarginal WM (SMWM); posterior thalamic radiation (PTR); inferior, middle and superior occipital WM (IOWM, MOWM, SOWM). | | | | | | | | |

**Supplemental Table 3: Final models evaluating the association between composite dMRI signatures and cognitive performance after accounting for amyloid burden, white matter hyperintensities (WMH), and demographics in the MCSA and ADNI cohorts. Models were corrected for multiple comparisons using Bonferroni correction across cognitive domains and predictors of interest within each cohort. An asterisk (*) indicates results that survived Bonferroni correction.** FA, fractional anisotropy; MD, mean diffusivity; HA, hypertensive arteriopathy; CAA, cerebral amyloid angiopathy (CAA); I, index.

|  | | **MCSA** | | |  | **ADNI** | | | |
| --- | --- | --- | --- | --- | --- | --- | --- | --- | --- |
|  |  | **Attention** | | **Memory** |  | **Attention** |  | **Memory** |  |
| dMRI Signature | Predictors | Estimate (S.E) | p-value | Estimate (S.E) | p-value | Estimate (S.E) | p-value | Estimate (S.E) | p-value |
| **HA-FAI model** |  | **Model R^2^ = 0.35)** | | **Model R^2^ = 0.30)** | | **Model R^2^ = 0.23)** | | **Model R^2^ = 0.37)** | |
|  | Intercept | 1.47 (0.31) | <0.001 | 1.05 (0.34) | 0.002 | -0.36 (0.34) | 0.289 | 0.56 (0.32) | 0.080 |
|  | Age | -0.04 (0.004) | <0.001 | -0.03 (0.01) | <0.001 | -0.001 (0.004) | 0.797 | -0.01 (0.004) | <0.001 |
|  | Sex | -0.33 (0.05) | <0.001 | -0.52 (0.06) | <0.001 | -0.04 (0.05) | 0.493 | -0.33 (0.05) | <0.001 |
|  | Education | 0.11 (0.01) | <0.001 | 0.13 (0.01) | <0.001 | 0.06 (0.01) | <0.001 | 0.08 (0.01) | <0.001 |
|  | Cycle number | 0.04 (0.02) | 0.038 | 0.13 (0.02) | 0.078 | - | - | - | - |
|  | HA-FAI | 0.14 (0.03) | <0.001***** | 0.03 (0.04) | 0.345 | 0.09 (0.03) | 0.002***** | 0.07 (0.03) | 0.007 |
|  | PIB | -0.87 (0.17) | <0.001 | -0.88 (0.19) | <0.001 | -0.01 (0.001) | <0.001 | -0.01 (0.001) | <0.001 |
|  | WMH | -0.03 (0.04) | 0.388 | -0.12 (0.04) | 0.009 | -0.04 (0.02) | 0.122 | -0.03 (0.02) | 0.165 |
| **HA-MDI model** |  | **(Model R^2^ = 0.34)** | | **(Model R^2^ = 0.30)** | | **Model R^2^ = 0.23)** | | **Model R^2^ = 0.38)** | |
|  | Intercept | 1.44 (0.32) | <0.001 | 1.00 (0.35) | 0.004 | -0.40 (0.34) | 0.247 | 0.48 (0.32) | 0.136 |
|  | Age | -0.04 (0.004) | <0.001 | -0.04 (0.01) | <0.001 | -0.001 (0.004) | 0.872 | -0.01 (0.004) | <0.001 |
|  | Sex | -0.32 (0.05) | <0.001 | -0.51 (0.06) | <0.001 | -0.03 (0.05) | 0.568 | -0.32 (0.05) | <0.001 |
|  | Education | 0.11 (0.01) | <0.001 | 0.13 (0.02) | <0.001 | 0.07 (0.01) | <0.001 | 0.08 (0.01) | <0.001 |
|  | Cycle number | 0.04 (0.02) | 0.043 | 0.13 (0.02) | 0.078 | - | - |  | - |
|  | HA-MDI | -0.09 (0.04) | 0.028 | -0.10 (0.04) | 0.048 | -0.09 (0.03) | 0.004***** | -0.08 (0.03) | 0.003***** |
|  | PIB | -0.86 (0.18) | <0.001 | -0.90 (0.19) | <0.001 | -0.01 (0.001) | <0.001 | -0.01 (0.001) | <0.001 |
|  | WMH | -0.06 (0.04) | 0.163 | -0.08 (0.05) | 0.086 | -0.04 (0.02) | 0.115 | -0.03 (0.02) | 0.179 |
| **CAA-FAI model** | | **(Model R^2^ = 0.34)** | | **(Model R^2^ = 0.30)** | | **Model R^2^ = 0.24)** | | **Model R^2^ = 0.37)** | |
|  | Intercept | 1.56 (0.32) | <0.001 | 1.12 (0.35) | 0.001 | -0.29 (0.33) | 0.380 | 0.72 (0.32) | 0.023 |
|  | Age | -0.04 (0.004) | <0.001 | -0.04 (0.01) | <0.001 | -0.002 (0.004) | 0.622 | -0.02 (0.004) | <0.001 |
|  | Sex | -0.31 (0.05) | <0.001 | -0.51 (0.06) | <0.001 | -0.02 (0.05) | 0.703 | -0.32 (0.05) | <0.001 |
|  | Education | 0.11 (0.01) | <0.001 | 0.13 (0.01) | <0.001 | 0.06 (0.01) | <0.001 | 0.08 (0.01) | <0.001 |
|  | Cycle number | 0.04 (0.02) | 0.054 | 0.13 (0.02) | <0.001 | - | - | - | - |
|  | CAA-FAI | 0.09 (0.03) | 0.006***** | 0.08 (0.04) | 0.031 | 0.09 (0.03) | 0.001***** | 0.02 (0.03) | 0.406 |
|  | PIB | -0.83 (0.18) | <0.001 | -0.86 (0.19) | <0.001 | -0.01 (0.001) | <0.001 | -0.01 (0.001) | <0.001 |
|  | WMH | -0.05 (0.04) | 0.236 | -0.08 (0.05) | 0.088 | -0.03 (0.02) | 0.170- | -0.04 (0.02) | 0.097 |
| **CAA-MDI model** | | **(Model R^2^ = 0.34)** | | **(Model R^2^ = 0.30)** | | **Model R^2^ = 0.22)** | | **Model R^2^ = 0.37)** | |
|  | Intercept | 1.55 (0.32) | <0.001 | 1.12 (0.34) | 0.001 | -0.21 (0.34) | 0.532 | 0.68 (0.32) | 0.034 |
|  | Age | -0.04 (0.004) | <0.001 | -0.04 (0.01) | <0.001 | -0.003 (0.004) | 0.429 | -0.02 (0.004) | <0.001 |
|  | Sex | -0.31 (0.05) | <0.001 | -0.49 (0.06) | <0.001 | -0.03 (0.05) | 0.531 | -0.32 (0.05) | <0.001 |
|  | Education | 0.11 (0.01) | <0.001 | 0.13 (0.01) | <0.001 | 0.07 (0.01) | <0.001 | 0.08 (0.01) | <0.001 |
|  | Cycle number | 0.04 (0.02) | 0.036 | 0.13 (0.02) | <0.001 | - | - | - | - |
|  | CAA-MDI | -0.10 (0.04) | 0.005***** | -0.11 (0.04) | 0.005***** | -0.04 (0.03) | 0.139 | -0.04 (0.03) | 0.169 |
|  | PIB | -0.84 (0.18) | <0.001 | -0.87 (0.19) | <0.001 | -0.01 (0.001) | <0.001 | -0.01 (0.001) | <0.001 |
|  | WMH | -0.04 (0.04) | 0.363 | -0.05 (0.05) | 0.271 | -0.04 (0.03) | 0.080 | -0.04 (0.02) | 0.126 |

**Supplemental Figure 1: Association of dMRI measurements with proxies of hypertensive arteriopathy (hypertension-[HTN]) and cerebral amyloid angiopathy (lobar cerebral microbleeds [CMB]) after adjusting for age and sex in Mayo Clinic Study of Aging (MCSA) participants without mixed CMBs.** Higher FA = better integrity (OR < 1, lower risk); Higher MD = worse integrity (OR > 1, higher risk). FA, fractional anisotropy; MD, mean diffusivity. An asterisk (*) represents regions that survived Bonferroni correction and the x axis is log-scaled.


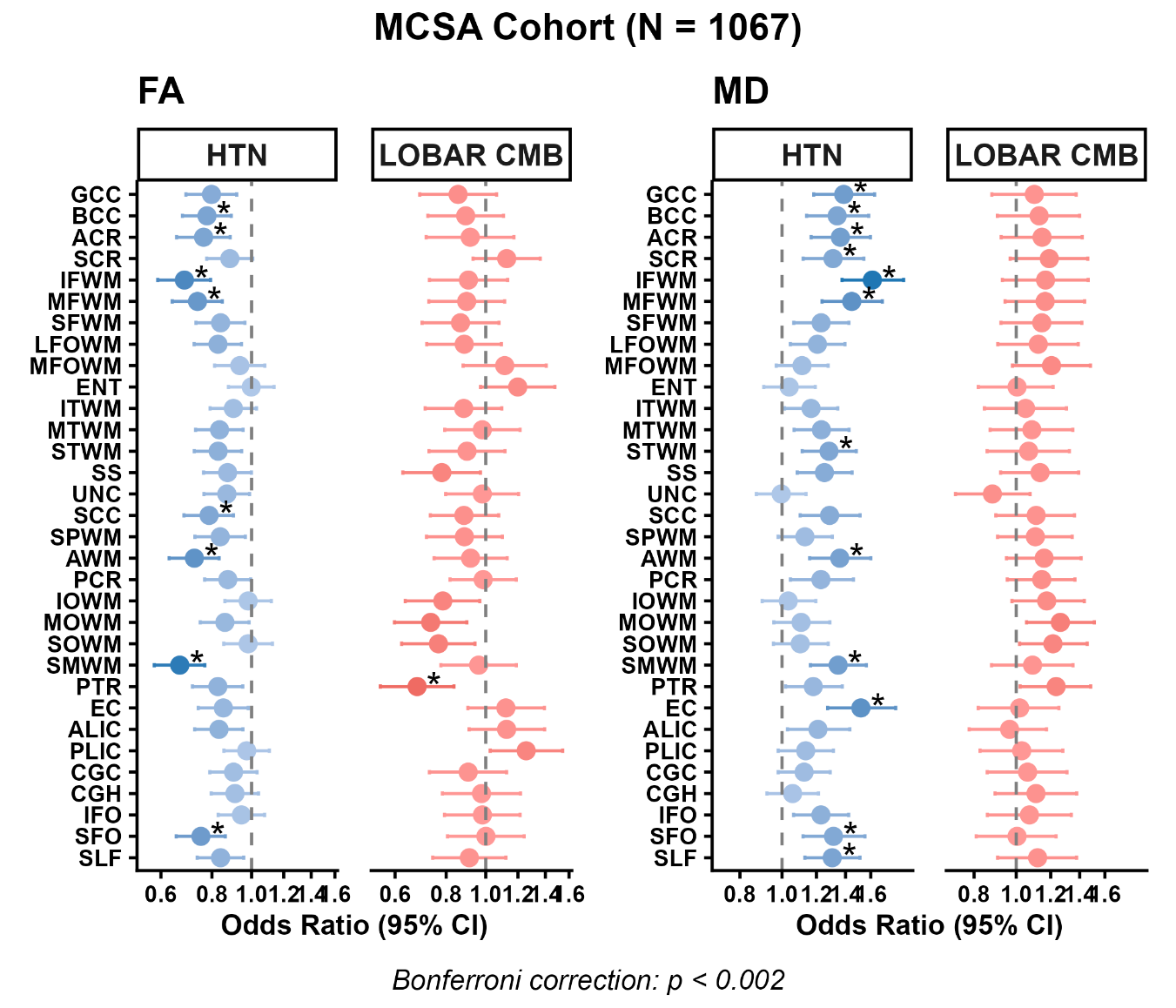


**Supplemental Figure 2: Heatmap illustrating tract-specific standardized estimates associated with proxies of hypertensive arteriopathy (hypertension: HTN) and cerebral amyloid angiopathy (lobar cerebral microbleeds [CMB]) (A). Scatterplots showing tract-level effect estimates in MCSA versus ADNI for FA and MD (B). Each point represents one tract; lines indicate linear fit with 95% confidence intervals.**

Tract-level effect estimates showed cross-cohort concordance between MCSA and ADNI for both FA and MD, with largely consistent effect directions but systematically attenuated effect sizes in ADNI.

**
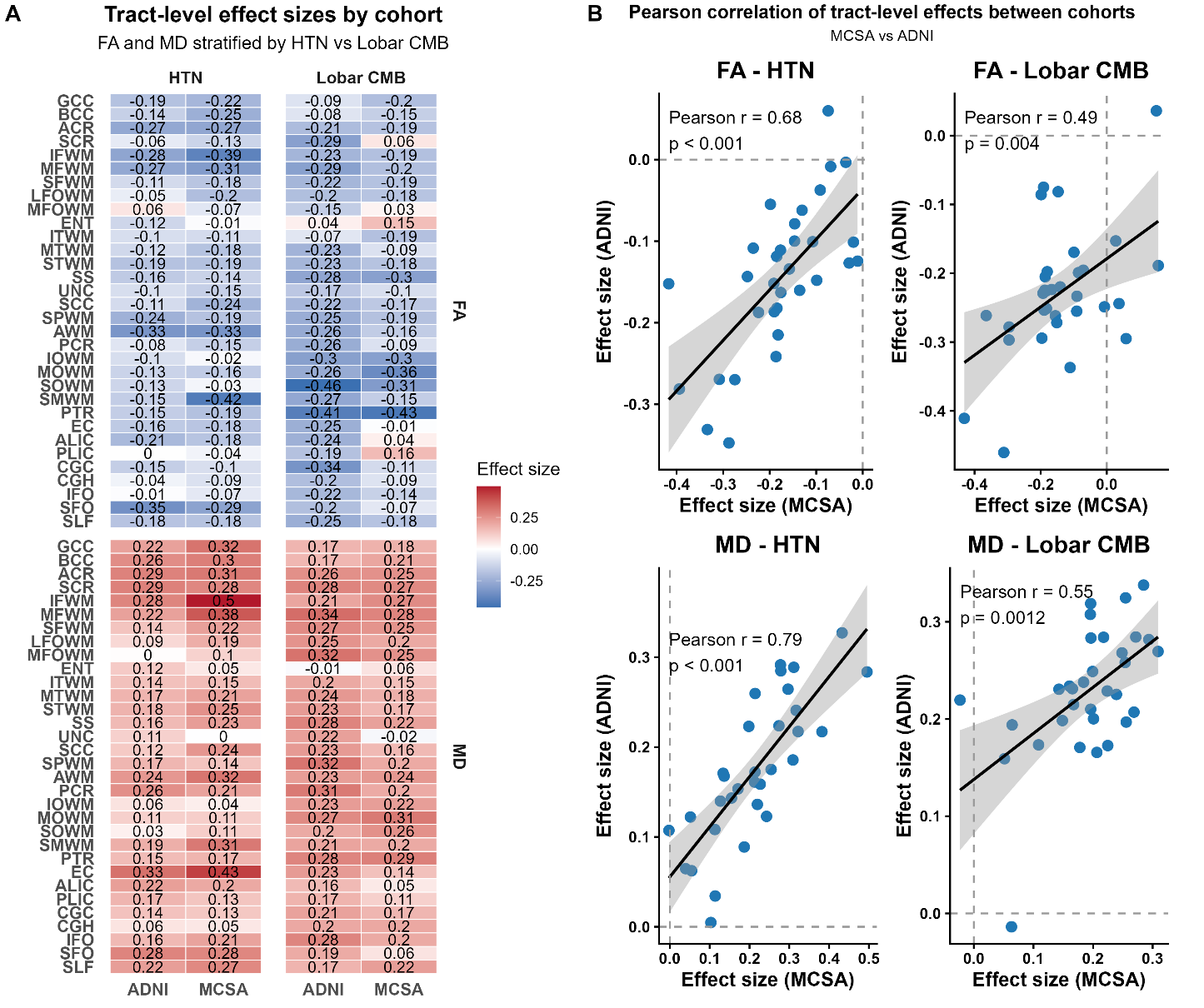
**

**Supplemental Figure 3: Association between composite dMRI signatures and cognitive performance in the Mayo Clinic Study of Aging (MCSA) (A) and the Alzheimer’s Disease Neuroimaging Initiative (ADNI) (B).** Model 1: Cognition ~ age + sex + education + cycle number + composite index; Model 2: Model 1 + amyloid PET. HA, hypertensive arteriopathy; CAA, cerebral amyloid angiopathy; FA, fractional anisotropy; MD, mean diffusivity; I‑index; MMSE, Mini‑Mental State Examination.


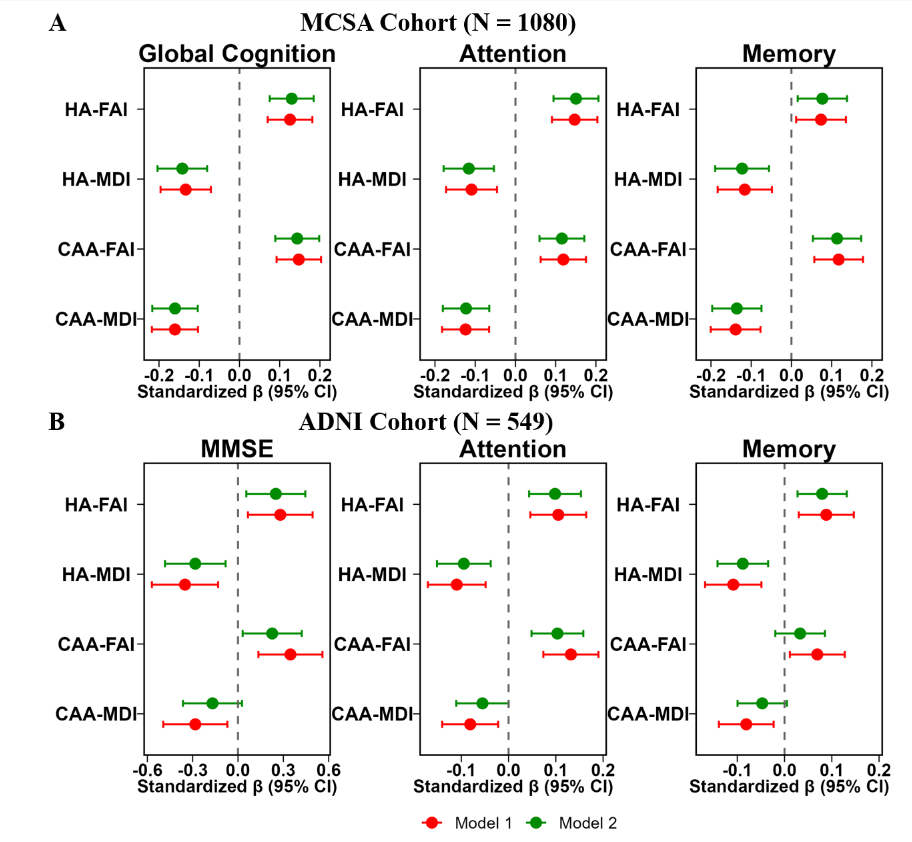

Supplement: Supplementary file 1 — Supplementary Material 1 [file 13024_2026_942_MOESM1_ESM.docx]
